# Inter-study and time-dependent variability of metabolite abundance in cultured red blood cells

**DOI:** 10.1186/s12936-021-03780-5

**Published:** 2021-07-02

**Authors:** Shivendra G. Tewari, Krithika Rajaram, Russell P. Swift, Bobby Kwan, Jaques Reifman, Sean T. Prigge, Anders Wallqvist

**Affiliations:** 1 Department of Defense Biotechnology High Performance Computing Software Applications Institute, Telemedicine and Advanced Technology Research Center, U.S. Army Medical Research and Development Command, Fort Detrick, MD USA; 2 The Henry M. Jackson Foundation for the Advancement of Military Medicine, Inc., Bethesda, MD USA; 3 Department of Molecular Microbiology and Immunology, Johns Hopkins University, Baltimore, MD USA; 4 Division of Biology and Biological Engineering, California Institute of Technology, Pasadena, CA USA

**Keywords:** Human red blood cells, Metabolic network modeling, Metabolism, Metabolomics, *Plasmodium falciparum*

## Abstract

**Background:**

Cultured human red blood cells (RBCs) provide a powerful ex vivo assay platform to study blood-stage malaria infection and propagation. In recent years, high-resolution metabolomic methods have quantified hundreds of metabolites from parasite-infected RBC cultures under a variety of perturbations. In this context, the corresponding control samples of the uninfected culture systems can also be used to examine the effects of these perturbations on RBC metabolism itself and their dependence on blood donors (inter-study variations).

**Methods:**

Time-course datasets from five independent studies were generated and analysed, maintaining uninfected RBCs (uRBC) at 2% haematocrit for 48 h under conditions originally designed for parasite cultures. Using identical experimental protocols, quadruplicate samples were collected at six time points, and global metabolomics were employed on the pellet fraction of the uRBC cultures. In total, ~ 500 metabolites were examined across each dataset to quantify inter-study variability in RBC metabolism, and metabolic network modelling augmented the analyses to characterize the metabolic state and fluxes of the RBCs.

**Results:**

To minimize inter-study variations unrelated to RBC metabolism, an internal standard metabolite (phosphatidylethanolamine C18:0/20:4) was identified with minimal variation in abundance over time and across all the samples of each dataset to normalize the data. Although the bulk of the normalized data showed a high degree of inter-study consistency, changes and variations in metabolite levels from individual donors were noted. Thus, a total of 24 metabolites were associated with significant variation in the 48-h culture time window, with the largest variations involving metabolites in glycolysis and synthesis of glutathione. Metabolic network analysis was used to identify the production of superoxide radicals in cultured RBCs as countered by the activity of glutathione oxidoreductase and synthesis of reducing equivalents via the pentose phosphate pathway. Peptide degradation occurred at a rate that is comparable with central carbon fluxes, consistent with active degradation of methaemoglobin, processes also commonly associated with storage lesions in RBCs.

**Conclusions:**

The bulk of the data showed high inter-study consistency. The collected data, quantification of an expected abundance variation of RBC metabolites, and characterization of a subset of highly variable metabolites in the RBCs will help in identifying non-specific changes in metabolic abundances that may obscure accurate metabolomic profiling of *Plasmodium falciparum* and other blood-borne pathogens.

**Supplementary Information:**

The online version contains supplementary material available at 10.1186/s12936-021-03780-5.

## Background

According to the 2019 World Health Organization (WHO) malaria report, there were 228 million cases and 405,000 deaths worldwide due to malaria [1]. The most lethal malaria parasite, *Plasmodium falciparum*, is associated with 99.7% of all cases and 93% of all malaria-associated deaths in the WHO African region [1]. During the symptomatic stage of malaria, *P. falciparum* infects red blood cells (RBCs) and undergoes asexual replication, eventually rupturing the RBCs and starting new infections. The RBC serves as the host cell, providing essential nutrients for *P. falciparum* growth during asexual multiplication and making it an integral component for ex vivo studies of *P. falciparum* for the symptomatic blood-stage of malaria.

The human body produces approximately 2 million RBCs every second [2], of which an average RBC can stay in the blood circulation for 100–120 days, giving rise to a distribution of young and old RBCs. Both young and old RBCs have differences in metabolism, e.g., young RBCs metabolize glucose at a rate that is 2.5 times the rate of old RBCs [3]. For the last few decades, synchronous cultures of parasite-infected RBCs (iRBCs) have been used to probe parasite biology with the ultimate aim of identifying more effective anti-malarial strategies. To perform these experiments, the parasites are propagated in RBCs collected from healthy blood donors. Therefore, for each independent experiment, malaria parasites replicate asexually in a different RBC environment that depends on the metabolic status of the blood donor.

Advances in high-resolution metabolomic methods allow the study of metabolic abundance alterations in hundreds of RBC metabolites upon parasite infection and any other perturbation. For example, studies have reported alterations in metabolite abundances of iRBC cultures due to drug treatments [4, 5] or nutrient deprivations [6, 7]. The influence of a perturbation on iRBC metabolism is typically reported with respect to changes observed in a “mock” culture of uninfected RBCs (uRBCs) maintained in parallel under identical culture conditions. However, the inter-study variability in RBC metabolism itself is unknown and assumed not to influence the analysis of parasite metabolism.

Herein, variability in abundances of metabolites was quantified in uRBC cultures of four independent studies that originally investigated blood-stage growth of the malaria parasites [7–10]. In addition, metabolomic data were collected from uRBC cultures maintained in experimental conditions akin to these four studies. In total, RBC metabolomic data were analysed from five independent studies using identical experimental methods and nearly identical culture media. To minimize any inter-study differences unrelated to RBC metabolism, an internal standard metabolite with minimal variation in abundance over time between the five datasets was identified that allowed us to consistently quantify inter-study variations. Metabolic network analyses were also performed to identify differences, if any, in the functional state of RBCs between the five datasets and compared them to the expected alterations in RBC metabolism due to parasite infection [11]. Not surprisingly, variation in abundances and fluxes of most RBC enzymes between the five different studies was not as substantial as the alterations caused in metabolic fluxes due to the parasite infection. Data are provided for all detected metabolites as well as a detailed evaluation of the expected variation in abundance of approximately 200 RBC metabolites that are consistently detected at all sampled time points and in all replicates with robust signals (> 1000 raw counts) in all five studies. In this set, a smaller subset showed substantial variation in metabolic fluxes associated with the highly variable metabolites. This latter subset can be used for comparisons in other studies to identify non-specific changes in metabolic abundances that may obscure accurate metabolomic profiling of *P. falciparum-*infected RBCs.

## Methods

### RBC experiments and data collection

Experiments were performed at 2% haematocrit in gassed flasks (94% N_2_, 3% O_2_, and 3% CO_2_) at 37 °C. O-positive human RBCs were obtained from healthy blood donors as part of Johns Hopkins University’s phlebotomy protocol (Institutional Review Board protocol number: NA_00019050). Previously described methods were used to deplete white blood cells from the collected blood [9], and RBCs were maintained in a culture medium that was originally designed to maintain continuous cultures of *P. falciparum* [9]. Quadruplicate samples were collected at 0, 8, 16, 24, 32, and 40 h after transfer into fresh culture medium. For all datasets described in this paper, sample collection began 2–3 days after blood was drawn. All samples were immediately spun down at 400×*g,* and flash froze 100 µL of pelleted RBCs and stored them at –80 °C. Then, quadruplicate samples were sent to Metabolon, Inc. (Durham, NC) for quantification of metabolites in the RBC pellets.

In addition, metabolomic data were analysed from four independent studies that originally examined *P. falciparum* metabolism [7–10]. These datasets were included in this analyses because the experiments performed in these studies use methods that are identical to this study and have only minor differences in their culture medium, making these datasets suitable for studying inter-study variations in RBC metabolism. As a perturbation standard, metabolomic data were also analysed from iRBC cultures that were maintained under normal conditions during blood-stage growth [9]. To ensure the robustness of the analyses, only metabolites with greater than 1000 raw counts at all sampled time points and across all the replicates of uRBC and iRBC cultures were included. Table 1 lists metadata of all metabolomic datasets that were analysed in this study. In Additional file 1, the raw metabolomic data collected during this study and the four independent studies are provided [7–10].

**Table 1:** Metabolomic studies of in vitro red blood cell culture systems originally designed to study *P. falciparum* intraerythrocytic development

Study	Label	Date of experiment^a^	RBC infection status	RPMI culture medium change^b^		References
				Action	Component	
1	Pure 1	4/2016	Uninfected	None	–	[9]
2	−Hxn	5/2017	Uninfected	Reduce to 0.5 μM	Hypoxanthine	[7]
3	+ Mev	3/2018	Uninfected	Add 50 μM	Mevalonate	[8]
4	+ Fos	11/2018	Uninfected	Add 1.0 μM	Fosmidomycin	[10]
5	Pure 2	5/2019	Uninfected	None	–	This study
6	iRBC	4/2016	Infected	None	–	[9]

^a^All experiments started within 2–3 days of blood collection. ^b^All experiments used Roswell Park Memorial Institute (RPMI)-1640 medium (Gibco, Gaithersburg, MD, USA) supplemented with 20 mM HEPES, 90 µM hypoxanthine, 0.3% sodium bicarbonate, 25 µg/mL gentamicin, 0.5 µM R-lipoic acid, and 0.5% AlbuMAX II (Life Technologies, Carlsbad, CA, USA)

+ *Fos* fosmidomycin-added RPMI medium, − *Hxn* hypoxanthine-limited RPMI medium, *iRBC* parasite-infected red blood cells, + *Mev* mevalonate-added RPMI medium, *Pure 1* pure RPMI medium, *Pure 2* pure RPMI medium, *RBC* red blood cells

### Global analyses of the data

As a first step, the raw data from each sample were normalized by its Bradford protein concentration, provided by Metabolon, Inc. (Additional file 1), and then quantile normalization was performed using the built-in MATLAB function “quantilenorm” to minimize batch-to-batch variability associated with data-extraction methods [12]. An internal-standard metabolite was then identified to mitigate inherent physiological variability associated with the donor’s metabolism, in addition to batch-to-batch variability associated with the data-extraction methods. In this study, total ion current (TIC) based methods were not employed to normalize the data because they tend to exaggerate the effect of metabolites with very high raw counts [13]. Moreover, TIC normalization assumes that most metabolites do not change under the tested experimental condition, but this assumption may not hold while making inter-study comparison**s** or comparing untreated RBCs to treated RBCs [13].

To identify an internal standard metabolite, the built-in MATLAB function “bootstrp” was used to generate 10,000 bootstrap samples from quadruplicate samples of all five RBC datasets for each metabolite and time point. Afterwards, a fold change with respect to a given time point *t* relative to 0 h (${\text{FC}}_{{0}}^{{\text{t}}}$) for each metabolite was computed using:

1 $$\begin{array}{*{20}c} {{\text{FC}}_{0}^{t} = \frac{1}{N}\mathop \sum \limits_{i = 1}^{N} \frac{{m_{t} }}{{m_{0} }}} \\ \end{array}$$

where *N* denotes the total number of bootstrap samples, and ${\text{m}}_{{0}}$ and ${\text{m}}_{{\text{t}}}$ represent abundance levels of metabolite *m* at 0 h and *t* h, respectively, of the experiment across the replicates of all five datasets.

The internal standard metabolite was identified using a metric $\zeta$, which is based on averaging ${\text{FC}}_{0}^{\text{t}}$ across all the time points (FC_0_) and the resulting standard deviation ($\upsigma$. Mathematically,

2 $$\begin{array}{*{20}c} {\zeta = \left| {\log_{2} {\text{FC}}_{0} } \right| + \frac{{\upsigma ^{2} }}{{{\text{FC}}_{0}^{2} }}} \\ \end{array}$$

The first term is a penalty term, while the second term measures variability in the metabolite with respect to the overall mean. The penalty term would increase $\zeta$ for metabolites that substantially increase/decrease over time, while the second term is the square of the coefficient of variation. We selected the internal standard metabolite based on the lowest value of $\zeta$.

Hierarchical clustering analysis (HCA) was performed using the built-in MATLAB function “clustergram,” with the Euclidean distance as a metric to cluster similar metabolites. The built-in MATLAB function “pca” was used to perform principal component analysis (PCA). To compute the rate of metabolic alteration (Δ*m*/Δ*t*) at a given time point *t*, the backward difference approximation of the first derivative was used:

3 $$\begin{array}{*{20}c} {\frac{\Delta m}{{\Delta t}} = \frac{{m_{t} - m_{t - 1} }}{\Delta t}} \\ \end{array}$$

Here, *m*_*t*_ and *m*_*t*-1_ denote the normalized abundance of metabolite *m* at time *t* and *t*-1, respectively, with *t* varying from 0 to 8, 16, 24, 32, and 40 h; Δ*m*/Δ*t* at 8, 16, 24, 32, and 40 h was computed. ∆*t* represents the difference between *t* and *t*-1.  

### Metabolic network analysis

To simulate the RBC metabolism, a proteomically-derived metabolic network model of a human RBC was used [14]. To integrate metabolomic data from uRBCs with the metabolic network model, the following assumptions were made: (1) flux through an enzyme is proportional to the amount of substrate and (2) a substrate with the lowest concentration becomes the rate-limiting step for reactions with multiple substrates. These assumptions hold true for substrates as well as products if a reaction is unidirectional. Therefore, the directionality of metabolic reactions was determined by identifying their thermodynamic feasibility, which is equivalent to analysing flux variability without any closed loop in the metabolic network [15]. This method was previously used to estimate RBC metabolism in response to hypoxanthine deprivation [7]. Briefly, the relative metabolite levels of substrates and products were used to scale basal unidirectional fluxes of the RBC and predict the temporal profile of RBC metabolism during the experiment. In Additional file 2: Text S1, all of the steps associated with the identification of the basal RBC metabolism, and integration of basal RBC metabolism and the time-resolved metabolomic data with the RBC model are provided.

## Results

In recent years, several studies collected metabolomic data from iRBC cultures in response to a wide range of perturbations [4, 7–9, 16]. Typically, the impact of a perturbation on metabolite abundances of iRBC culture is quantified relative to their abundance in a parallel uRBC culture maintained under identical conditions. However, this type of comparison is susceptible to false discoveries if the perturbation, via specific or non-specific mechanisms, causes significant alterations in metabolite abundances of the uRBC culture itself. Moreover, metabolic alterations cannot ideally be compared from one study to another because inter-study variability in the abundance of RBC metabolites is largely unknown. Therefore, in this study, the objective was to characterize inter-study variability in the abundance of commonly detected RBC metabolites. First, the datasets were analysed and an internal standard metabolite was identified. Then, variability in metabolites of uRBC cultures was characterized and compared with expected variability in a representative iRBC culture—a perturbation standard. Lastly, the data were integrated with an erythrocytic metabolic network model to identify core RBC metabolism carrying the majority of metabolic flux during each independent experiment.

### Global analyses of the data

**Fig. 1:**
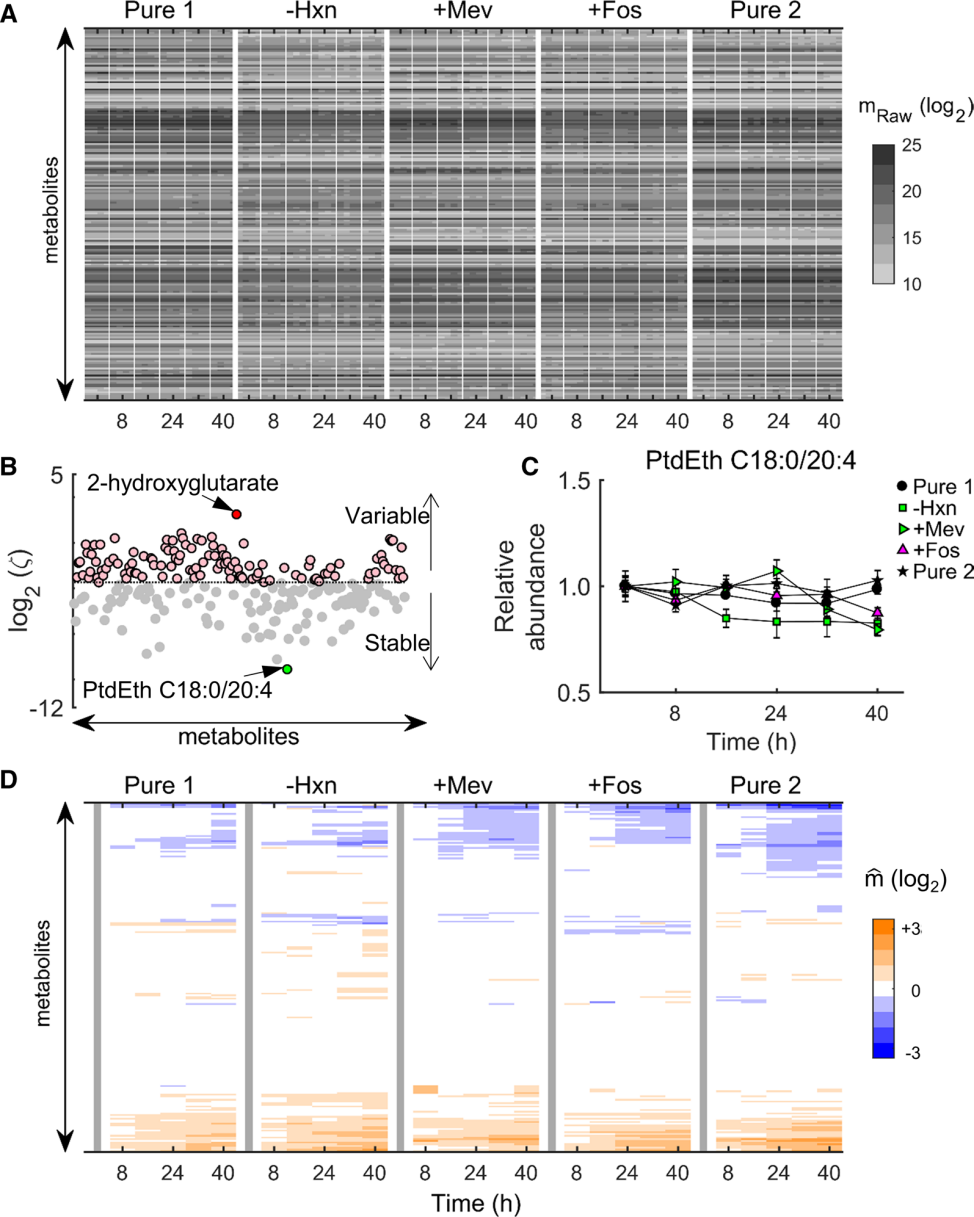
Overview of metabolomic data from uninfected red blood cells maintained under near-identical culture conditions. **A** Raw counts of metabolites (m_Raw_) that were common between the five independent experiments (Pure 1, −Hxn, + Mev, + Fos, and Pure 2). The thin vertical lines separate quadruplicate samples at each time point, while the thick vertical lines separate the five studies. The abscissa denotes the time point of sample collection during an experiment; the ordinate represents the different metabolites. **B** Estimated $\zeta$ values for all overlapping metabolites. The coloured markers denote the following: pink, metabolites with $\zeta \ge \stackrel{{\sim }}{\zeta}$; gray, metabolites with $\zeta< \stackrel{{\sim }}{\zeta}$; green, internal standard metabolite (PtdEth C18:0/20:4); red, an outlier metabolite (2-hydroxyglutarate). The dotted line is showing $\stackrel{{\sim }}{\zeta}$, the median value of $\zeta$ for all the metabolites. **C** The temporal profile (relative to 0 h) of the internal standard metabolite during the five experiments. **D** The average of raw metabolomic data in **A** after normalization with the internal standard and raw counts at 0-h time point under each experiment. We computed the average using the normalized values of each metabolite in the quadruplicate samples at each time point of every culture condition. $\widehat{\text{m}}$ denotes the time-resolved abundance of metabolite *m* after the normalization and the averaging.  + Fos, fosmidomycin-added RPMI medium; -Hxn, hypoxanthine-deprived RPMI medium; + Mev, mevalonate-added RPMI medium; PtdEth, phosphatidylethanolamine; Pure 1, pure RPMI medium; Pure 2, pure RPMI medium; RPMI, Roswell Park Memorial Institute

To facilitate inter-study data comparisons, an internal standard metabolite was identified to minimize inter-study differences arising due to technical variations, and then the normalized data were analysed to identify variability in the abundance of commonly detected RBC metabolites. For the analysis, only metabolites that had greater than 1000 raw counts at all the time points of each quadruplicate sample maintained under each culture condition were included. Figure 1A shows the raw metabolomic data (m_Raw_) obtained from the five independent experiments that maintained RBCs under near-identical culture conditions. The culture media used in these experiments are listed in Table 1. To identify the internal standard metabolite, the average fold change in metabolite abundance over time relative to 0 h (FC_0_) was computed for all the replicates across all the culture media and a metric ζ penalizing metabolites with highly variable abundance over time was used (see Methods). Figure 1B shows ζ values of metabolites that met the raw count criterion across all the time points of all the studies. Phosphatidylethanolamine (PtdEth C18:0/20:4) underwent minimal alterations, and 2-hydroxyglutarate experienced maximal alterations over time (see Additional file 3 for ζ values of all metabolites). Figure 1C illustrates the relative abundance of the internal standard across different culture conditions. Because PtdEth C18:0/20:4 was the least varying metabolite based on the probability distribution of ζ (Additional file 2: Fig. S1), this metabolite was used to normalize the raw metabolomic data and minimize any inter-study variations arising due to day-to-day variations [17]. Figure 1D illustrates averaged metabolomic data after normalization with the 0-h time point and the identified internal standard. $\widehat{\text{m}}$ was used to denote the time-resolved abundance of metabolite *m* after the normalization and averaging across the quadruplicates. After normalization, similar trends of increasing (or decreasing) metabolite abundances across the five datasets emerged (Fig. 1D), which were not visible in the raw data (Fig. 1A).

**Fig. 2:**
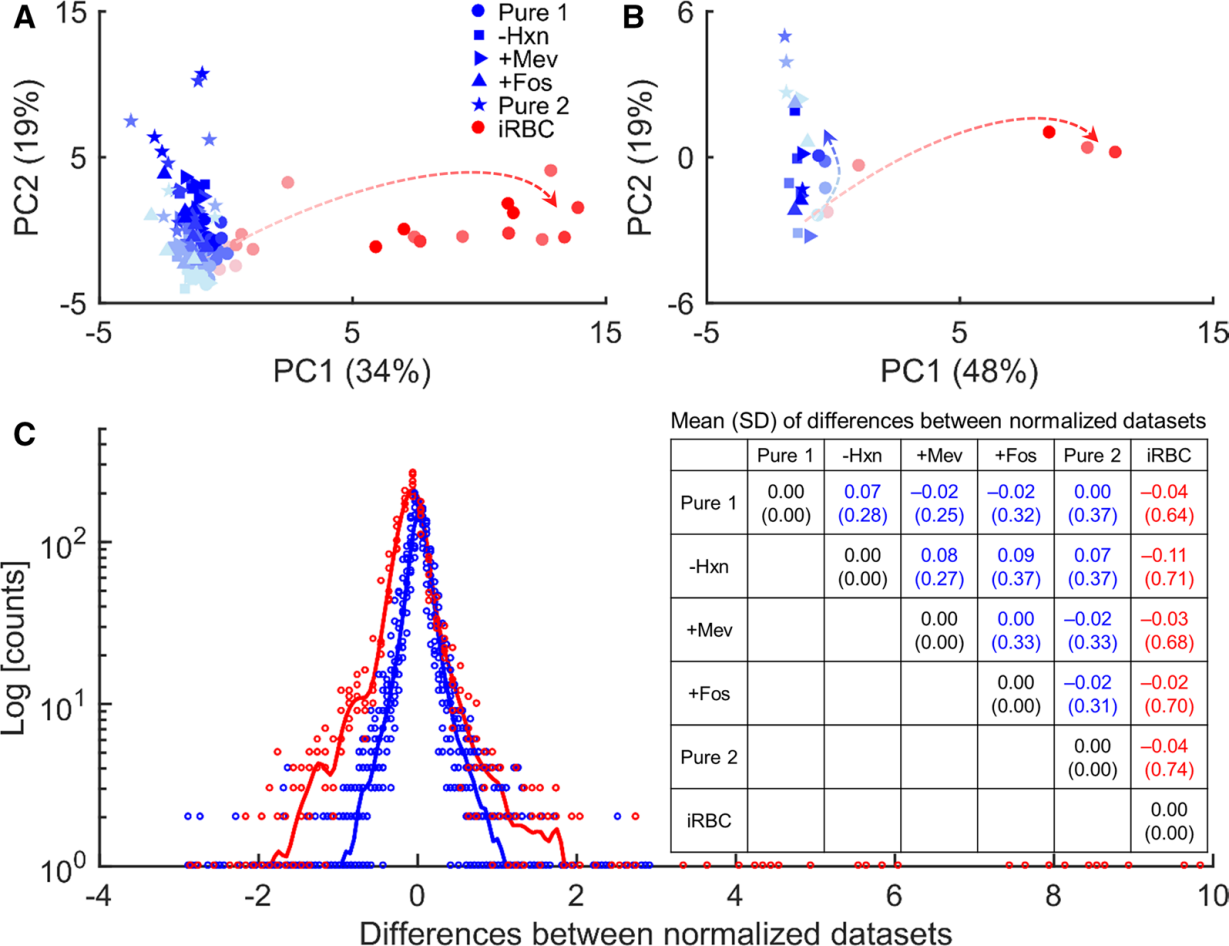
Inter-study variation in metabolomic data from uninfected red blood cells (uRBC) of the five studies and parasite-infected red blood cells (iRBC). **A** Principal component analysis (PCA) of metabolomic data after normalization with the 0-h time point and the identified internal standard. For each time point, there are four data points corresponding to the quadruplicate samples of each study. **B** PCA of averaged metabolomic data after normalization with the 0-h time point and the identified internal standard. The red arrows denote separation of iRBC data from the uRBC as time progresses, indicated by light to dark red, while the blue arrow denotes separation within the uRBC data. Abscissa and ordinate in A and B denote the percentage of the total data variance explained by each principal component. **C** Raw differences between normalized data from uRBC cultures and the iRBC culture. The difference was taken within uRBC data for each time point (blue circles) or between uRBC data and iRBC data for each time point (red circles). The table in the inset shows the average differences. Note that for PCA and difference computations, we did not include the 0-h data, as they serve as the comparator for other time points. + Fos, fosmidomycin-added RPMI medium; -Hxn, hypoxanthine-deprived RPMI medium; + Mev, mevalonate-added RPMI medium; PC1, first principal component; PC2, second principal component; Pure 1, pure RPMI medium; Pure 2, pure RPMI medium; RPMI, Roswell Park Memorial Institute; SD, standard deviation

To gauge the impact of a study-specific perturbation, such as parasite infection, on RBC metabolism, we performed PCA of the normalized data from the uRBC cultures while including metabolomic data from the representative iRBC culture (Table 1). Prior to performing the PCA, the raw metabolomic data from iRBC cultures were normalized using methods akin to Fig. 1D and then the data were averaged across the replicates at each time point. Figure 2A, B, show the first two principal components of metabolomic data from uRBC (blue) and iRBC (red) cultures before (A) and after performing the averaging (B) across the replicates. Both quadruplicate and averaged data from uRBC and iRBC cultures separate along the two components, highlighting gross differences between the two conditions that increase with time (red arrow, Fig. 2, A and B). To quantify the degree of similarity (or dissimilarity) between datasets, differences in normalized values of the metabolites over time were computed between different studies and parasite infection. Figure 2C shows the spread of variability between metabolic abundances, irrespective of the time points, for data from uRBC (blue circles) and iRBC (red circles) cultures. The solid blue and red lines are fits to differences shown with blue circles and red circles, respectively. Overall, the differences between datasets from the uRBC cultures are less than the differences between datasets from uRBC and iRBC cultures, indicating that most metabolite abundances in uRBC cultures changed minimally over time irrespective of the culture medium.

### Changes in uRBC metabolites over time

Next, specific metabolite abundances that vary substantially over time across uRBC cultures were identified. Specifically, the average of normalized metabolite abundances across all five datasets was computed to identify substantially varying metabolites. Table 2 lists metabolites that increased substantially across all datasets at all time points relative to 0 h. There was an approximately two-fold increase in the abundance of metabolites involved in the synthesis of glutathione, namely α-ketoglutarate [18], ophthalmate, 5-oxoproline, and glutamate (Table 2). There was also a similar increase in glycolysis products, namely fructose, 3-phosphoglycerate, sedoheptulose-7-phosphate, and lactate (Table 2), which play an important role in the synthesis of nicotinamide adenine dinucleotide phosphate (NADPH), the primary reducing equivalent in the RBCs [19].

**Table 2:** Metabolites that vary substantially across all datasets at all time points relative to 0 h

Major pathway	Subordinate pathway	Metabolites	Mean (SD)
Amino acids	Glutamate metabolism	Glutamate	1.74 (0.48)
		Glutamate, γ-methyl ester	1.54 (0.33)
	Glutathione metabolism	Ophthalmate	1.96 (0.58)
		5-Oxoproline	1.92 (0.54)
Carbohydrates	Fructose, mannose, and galactose metabolism	Mannitol/sorbitol	1.99 (0.59)
		Fructose	1.69 (0.54)
	Glycolysis, gluconeogenesis, and pyruvate metabolism	Lactate	2.41 (1.26)
		3-Phosphoglycerate	1.76 (0.40)
		Phosphoenolpyruvate	1.71 (0.27)
	Pentose phosphate pathway	Sedoheptulose-7-phosphate	2.93 (1.16)
Cofactors and vitamins	Haemoglobin and porphyrin metabolism	Heme	1.83 (0.41)
	Vitamin B6 metabolism	Pyridoxal	2.41 (0.74)
Energy	Tricarboxylic acid cycle	α-Ketoglutarate	2.19 (0.87)
Lipid	Endocannabinoid	Oleoyl ethanolamide	2.22 (1.09)
		Stearoyl ethanolamide	1.75 (0.44)
	Long-chain fatty acid	Erucate (22:1n9)	1.72 (0.44)
Peptide	Gamma-glutamyl amino acid	γ-Glutamylisoleucine^**‡**^	2.33 (0.72)
		γ-Glutamylleucine	2.14 (0.59)
		γ-Glutamylthreonine	2.10 (0.73)

Metabolites that have $\stackrel{\mathrm{-}}{\text{m}}$ greater than 1.5. Here, $\stackrel{\mathrm{-}}{\text{m}}$ represents average value of $\widehat{\text{m}}$ (shown in Fig. 1D). The average was taken across all datasets and time points (except 0 h). ^**‡**^Metabolite identified based on m/z ratio alone with no external standard for validation

The RBCs carry oxygen with the help of haemoglobin, which contains iron, making haemoglobin prone to oxidation. The RBC manages oxidative stress with the help of glutathione and reducing equivalents. Therefore, an accumulation of metabolites involved in glutathione synthesis and NADPH suggests that RBCs invoke antioxidant processes to maintain low levels of oxidative stress under in vitro conditions. It is known that oxidation of haemoglobin produces methaemoglobin [19], which the RBCs degrade via pathways that do not require ATP or ubiquitin [20]. In concordance with these reports, an approximately two-fold increase in peptides and haem in RBCs across all the cultures was found (Table 2). These results suggest that, under in vitro conditions, degradation of oxidized haemoglobin occurs at a rate that is proportional to glycolysis in each independent experiment.

**Fig. 3:**
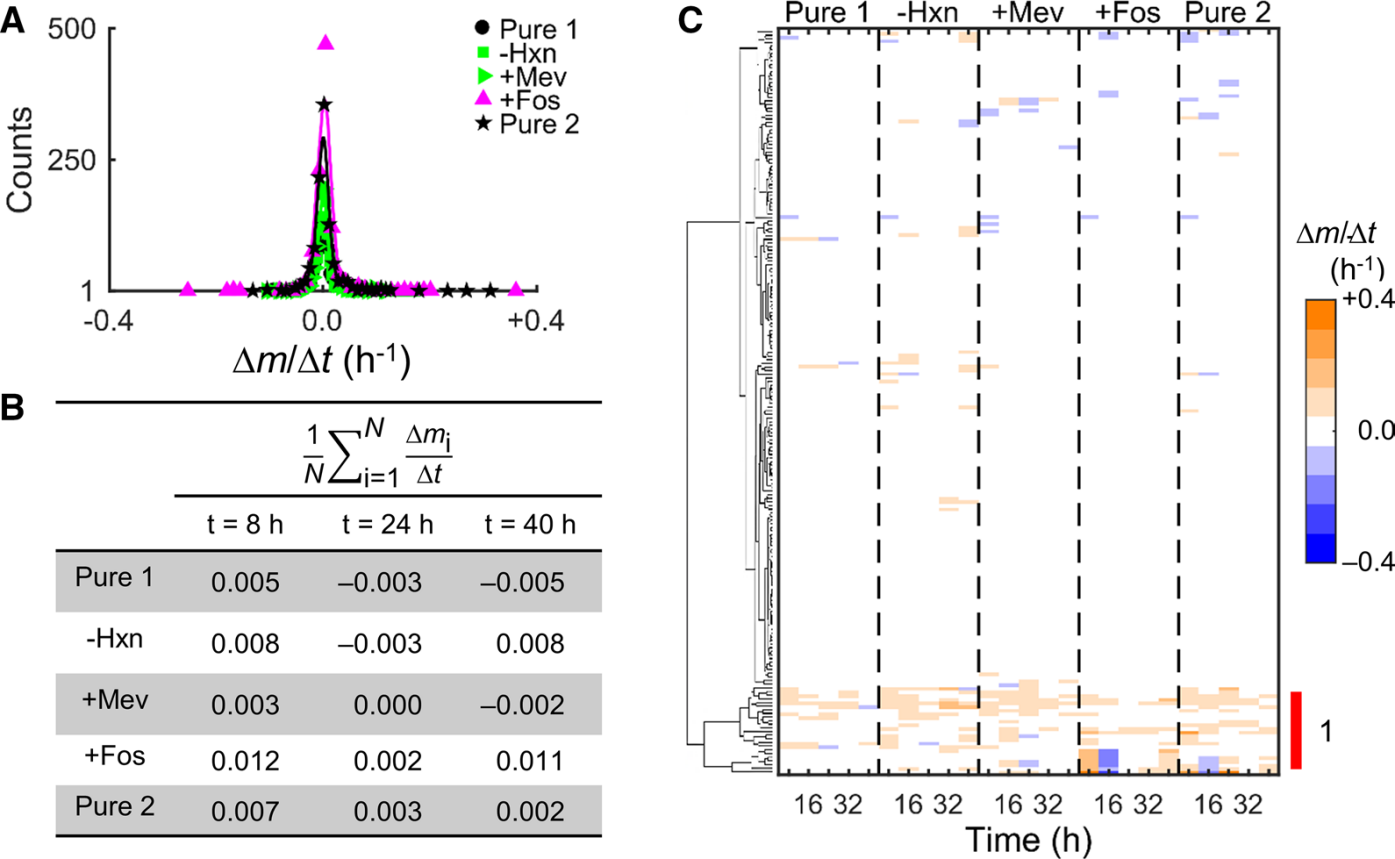
Rates of change in metabolite abundances (Δ*m*/Δ*t*) of red blood cells during the five studies. **A** Counts of metabolites within the range of Δ*m*/Δ*t* shown on the abscissa between 8 and 40 h of each experiment. **B** Average rate of change at the indicated time points of each experiment, where *N* denotes the total number of metabolites. **C** Hierarchical clustering of Δ*m*/Δ*t* across the five studies, highlighting the cluster (annotated with the number 1) with noticeably different Δ*m*/Δ*t* values. Note that we have computed Δ*m*/Δ*t* at 8, 16, 24, 32, and 40 h of the experiment (see Methods). + Fos, fosmidomycin-added RPMI medium; -Hxn, hypoxanthine-deprived RPMI medium; + Mev, mevalonate-added RPMI medium; Pure 1, pure RPMI medium; Pure 2, pure RPMI medium; RPMI, Roswell Park Memorial Institute

**Fig. 4:**
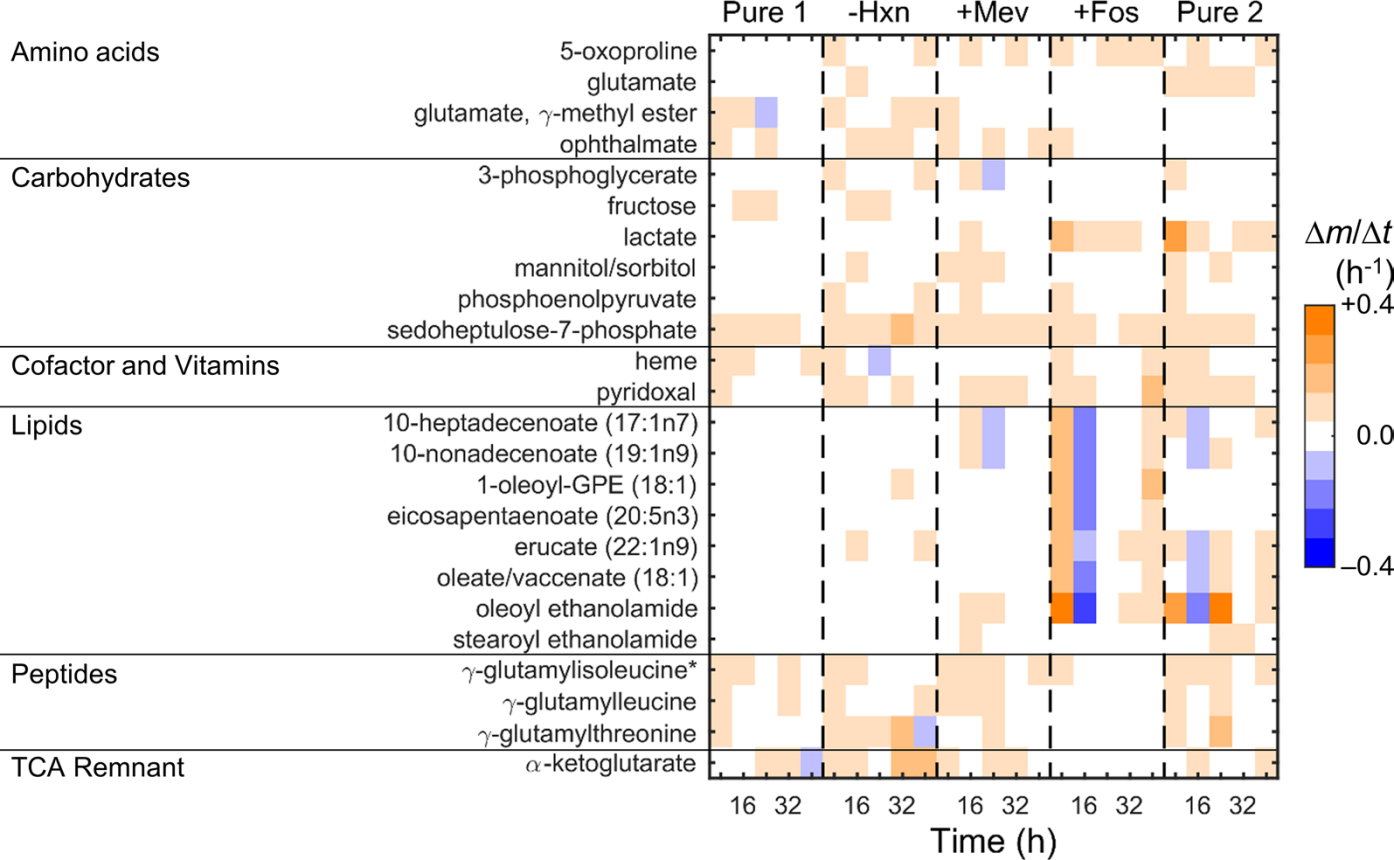
Metabolites with a noticeably different rate of change (Δ*m*/Δ*t*) in abundance in the five studies. We have categorized the metabolites in Cluster 1 of Fig. 3C based on their specific metabolite classes. + Fos, fosmidomycin-added RPMI medium; GPE, glycerophosphoethanolamine; -Hxn, hypoxanthine-deprived RPMI medium; + Mev, mevalonate-added RPMI medium; Pure 1, pure RPMI medium; Pure 2, pure RPMI medium; RPMI, Roswell Park Memorial Institute; TCA, tricarboxylic acid

To further identify highly variable metabolites in uRBC cultures, we computed the rate of change in metabolic abundances (Δ*m*/Δ*t*). Figure 3A shows the distribution of Δ*m*/Δ*t* at each time point under each culture medium. Overall, most metabolites have a near-zero rate of change, emphasizing that most metabolites do not change noticeably over time. Figure 3B shows the average of Δ*m*/Δ*t* at specific time points in each independent experiment, again illustrating that most metabolites change minimally over time. To identify specific metabolites with significant rates of change, we performed HCA of metabolic rate of change (Fig. 3C). HCA resulted in identification of a cluster (annotated with the number 1) containing metabolites that appeared to be changing noticeably in each independent culture experiment as compared to other metabolites. Figure 4 categorizes metabolites in Cluster 1 based on their respective metabolic classes. In addition to the metabolites listed in Table 2, this analysis also identified five additional lipid metabolites, namely 10-heptadecenoate (17:1n7), 10-nonadecenoate (19:1n9), 1-oleoyl-glycerophosphoethanolamine (18:1), eicosapentaenoate (20:5n3), and oleate/vaccenate (18:1). The appearance of metabolites listed in Table 2 during this analysis (Fig. 4) suggests that these metabolite abundances indeed vary substantially within the RBCs irrespective of the culture medium and any study-specific perturbation, such as parasite infection. Until now, the analyses in this study focused on alterations in abundance of specific metabolites; however, RBC metabolism depends on an inter-connected network of metabolic enzymes, converting one metabolite into another. Therefore, to quantify the impact of these alterations in metabolite abundances on the RBC metabolism, an in silico model of RBC metabolism was utilized [14]—presented in the next subsection.

### Model predicted metabolic state of RBC

**Fig. 5:**
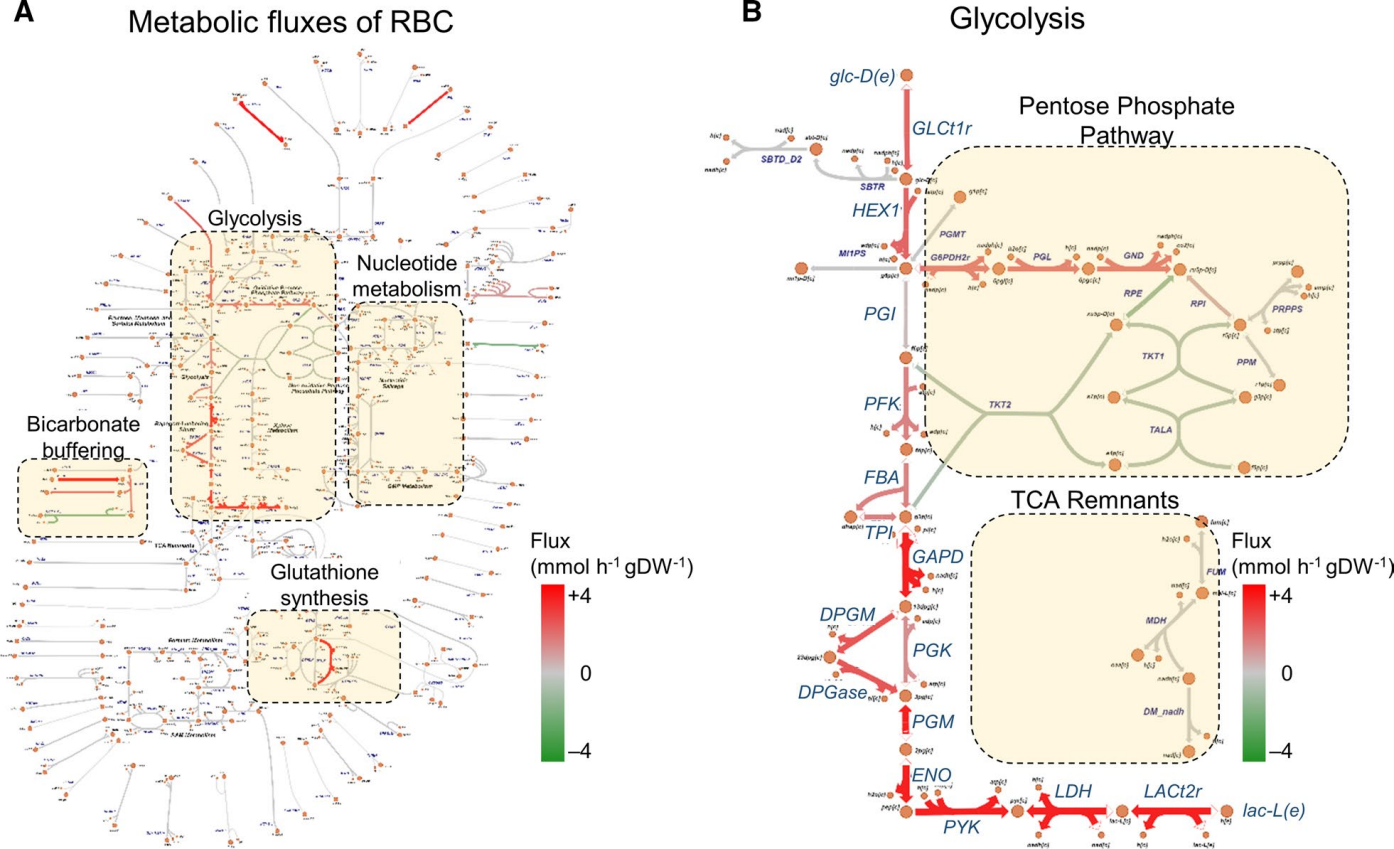
Overview of estimated metabolic fluxes at 0 h in red blood cells (RBC) maintained under pure Roswell Park Memorial Institute culture medium. **A** Model-predicted RBC fluxes at 0 h have been overlaid on a drawing of the RBC metabolic network [21] using the Escher web tool [22]. The shaded boxes highlight glycolysis, bicarbonate buffering, nucleotide metabolism, and glutathione synthesis in the RBC. **B** Detailed view of RBC enzyme fluxes at 0 h converting glucose into lactate. The shaded boxes highlight the pentose phosphate pathway and remnant reactions of the tricarboxylic acid (TCA) cycle. The orange circles denote metabolites, and thick/thin lines represent enzymes. The line thickness is proportional to the magnitude of enzymatic flux. DPGM, diphosphoglyceromutase; DGPase, diphosphoglycerate phosphatase; ENO, enolase; FBA, fructose bisphosphate aldolase; GAPD, glyceraldehyde 3-phosphate dehydrogenase; gDW, gram dry weight of RBC; glc-D(e), medium d-glucose; GLCt1r, glucose transporter; HEX1, hexokinase; lac-L(e), medium l-lactate; LACt2r, l-lactate reversible transport via proton symport; LDH, l-lactate dehydrogenase; PFK, phosphofructokinase; PGI, glucose 6-phosphate isomerase; PGK, phosphoglycerate kinase; PGM, phosphoglycerate mutase; PYK, pyruvate kinase; TCA, tricarboxylic acid; TPI, triose-phosphate isomerase

To gain insight into the functional state of RBC metabolism during the five studies, the metabolomic data from the respective studies were used to estimate the corresponding RBC metabolic fluxes. Figure 5A illustrates metabolic fluxes at 0 h for RBCs maintained under pure RPMI culture medium, labeled “Pure 1” in Table 1. In Additional file 2: Figs. S2–S30, an overview of RBC metabolic states at 8, 16, 24, 32, and 40 h and at every sampled time point of the other four studies is provided. The Escher web tool [22] and the RBC map drawn by Buchweitz et al. [21] were used to visualize the RBC model simulations of this study. It was determined that the glycolysis pathway carried fluxes of high magnitude in the RBC network. In addition, enzymes of the bicarbonate buffering system and glutathione oxidoreductase (GTHO) also exhibited substantial metabolic flux. The map boundary shows transport reactions (Fig. 5A), of which the only non-glycolytic reactions that appeared significant were those containing sodium and potassium ions. These results highlight the dependence of RBCs on glucose for generation and storage of high-energy phosphates [23].

Figure 5B provides a detailed view of the glycolysis pathway to further shed light on the involved glycolytic enzymes. In this representation, orange circles denote metabolites, and thick/thin lines represent the metabolic enzymes. The thickness and colour of each line are proportional to the metabolic enzyme flux shown in the colour map. The pentose phosphate pathway (PPP), which generates reducing equivalents used by antioxidant processes, such as GTHO, is highlighted. It was determined that the majority of the glucose is diverted to PPP, which then reenters glycolysis via phosphofructokinase (Fig. 5, PFK) and glyceraldehyde 3-phosphate dehydrogenase (Fig. 5, GAPD) to facilitate the synthesis of 2,3-bisphosphoglyceric acid (2,3-BPG) via diphosphoglyceromutase (Fig. 5, DPGM). The 2,3-BPG binds oxygenated and deoxygenated haemoglobin and facilitates the transport of oxygen to the tissues [24]. Eventually, lactate dehydrogenase (Fig. 5, LDH) converts the glucose carbons into lactate, which is transported out of the RBC via a lactate transporter (Fig. 5, LACt2r).

To identify metabolic fluxes that vary substantially over time, the study focused on metabolic fluxes having flux differences between a pair of studies that is greater than flux differences in 95% of the corresponding metabolic fluxes. To this end, all unique combinations (^5^C_2_ = 10) of the five model simulations, corresponding to the five independent datasets, were compared to quantify variability in estimated metabolic fluxes (Additional file 4). It was determined that approximately 95% of the metabolic fluxes varied less than 0.1 (millimole per hour per gram dry weight of RBC) between any pair of two studies. These results suggest that the majority of enzymatic fluxes do not vary substantially over time in each independent experiment, an observation that is consistent with the metabolomic data (Figs. 2 and 3). Table 3 lists the metabolic enzymes that vary substantially over time. To quantify the intra-study variability in these metabolic fluxes, the flux span of these enzymes in each study (Table 3) was computed. By performing this additional computation, it was established that these metabolic fluxes varied substantially within each and every study condition.

**Table 3:** Model-predicted metabolic enzymes that vary substantially over time

Metabolic pathway	Metabolic reaction name	Absolute flux span (mmol h^−1^ gDW^−1^)^‡^				
		Pure 1	-Hxn	+ Mev	+ Fos	Pure 2
Bicarbonate buffer system	Bicarbonate transport (${\text{C}}{\text{l}}^{-}\text{/HC}{\text{O}}_{3}^{-}$ exchange)	0.45	0.53	0.65	0.27	0.81
	Carbonic anhydrase	0.45	0.53	0.65	0.27	0.81
Glutamate metabolism	Glutathione oxidoreductase	0.91	1.06	1.30	0.53	1.61
Glycolysis	Enolase	1.19	0.53	0.87	0.60	1.57
	Glyceraldehyde-3-phosphate dehydrogenase	1.19	0.53	0.87	0.60	1.57
	Glucose transport (uniport)	0.46	0.28	0.55	0.27	0.87
	l-Lactate reversible transport via proton symport	1.19	0.53	0.87	0.60	1.57
	l-Lactate dehydrogenase	1.19	0.53	0.87	0.60	1.57
	Phosphoglycerate kinase	1.19	0.53	0.87	0.60	1.57
	Phosphoglycerate mutase	1.19	0.53	0.87	0.60	1.57
	Pyruvate kinase	1.19	0.53	0.87	0.60	1.57
	Hexokinase (d-glucose:ATP)	0.46	0.28	0.55	0.27	0.87
	Triose-phosphate isomerase	0.73	0.25	0.35	0.27	0.68
Ion transport	K^+^-Cl^–^ cotransport	0.45	0.53	0.65	0.27	0.81
	H^+^ exchange	0.36	0.13	0.71	0.27	1.41
	Na^+^/K^+^ ATPase	0.92	0.25	0.45	0.25	0.85
Oxidative phosphorylation	Inorganic diphosphatase	0.29	0.15	0.32	0.14	0.30
Pentose phosphate pathway	Glucose 6-phosphate dehydrogenase	0.46	0.53	0.65	0.27	0.81
	Phosphogluconate dehydrogenase	0.46	0.53	0.65	0.27	0.81
	6-Phosphogluconolactonase	0.46	0.53	0.65	0.27	0.81
	Ribulose 5-phosphate 3-epimerase	0.31	0.35	0.42	0.20	0.56

A metabolic enzyme with a flux value that differs by more than 0.1 mmol per hour per gram dry weight (gDW) of red blood cell (RBC) for at least four time points between two studies is considered a substantially variable metabolic flux. Approximately 95% of the metabolic fluxes had a standard deviation of less than 0.1 mmol per hour per gram dry weight of RBC between two study conditions. ^**‡**^The absolute difference between the maximum and minimum metabolic flux value for the simulated duration of the experiment. We have provided the maximum and minimum values of each metabolic enzyme in Additional file 4

To ascertain the significance of the variations identified in these metabolic fluxes, a cross-comparison of the top five high-magnitude fluxes in the RBCs with their values under each study condition and in the iRBC dataset was performed. Table 4 lists the average of absolute differences in GTHO fluxes between all possible unique pair-wise comparisons (^6^C_2_ = 15). The absolute differences of GTHO fluxes between different uRBC studies were not as large as those within the iRBC study [11]. In fact, the absolute differences of all five high-magnitude fluxes between different uRBC studies were not as large as those within the iRBC study (Additional file 5: Tables S1–S5). These results suggest that the variations occurring in metabolic fluxes of uRBCs over time do not overshadow the perturbations of parasite infection in the case of these high-magnitude fluxes; but in the case of RBC enzymes that vary substantially over time (Table 3), this may not be the case. Therefore, fluxes of the most variable RBC enzymes, listed in Table 3, were also compared with their fluxes in the iRBC study. An inter-study comparison, similar to Table 4, was performed, and it was determined that the influence of parasite infection on most of the enzyme fluxes, except inorganic diphosphatase (PPA), was substantial as compared to their inter-study flux variability in the uRBC studies (Additional File 6). The model-predicted PPA flux varied substantially between different uRBC studies and was comparable with its flux in the iRBC study (Additional File 6: Table S15). Therefore, during inter-study comparisons, the blood-batch variability in the PPA flux can obscure the effects of parasite infection on the RBC-PPA activity.

**Table 4:** Average (SD) of absolute differences in glutathione oxidoreductase (GTHO) fluxes over time between different conditions and parasite infection

	Pure 1	-Hxn	+ Mev	+ Fos	Pure 2	iRBC^a^
Pure 1	0.00	0.34	0.46	0.20	0.43	2.54
	(0.00)	(0.24)	(0.34)	(0.21)	(0.38)	(0.31)
−Hxn		0.00	0.20	0.24	0.54	2.62
		(0.00)	(0.22)	(0.19)	(0.39)	(0.37)
+ Mev			0.00	0.26	0.50	2.66
			(0.00)	(0.24)	(0.38)	(0.49)
+ Fos				0.00	0.34	2.67
				(0.00)	(0.37)	(0.21)
Pure 2					0.00	2.45
					(0.00)	(0.58)
iRBC						0.00
						(0.00)

^a^We used the published value of GTHO flux estimated in parasite-infected RBC (iRBC) [11] to perform this comparison. *–Hxn* hypoxanthine-deprived RPMI medium, + *Mev* mevalonate-added RPMI medium, + *Fos* fosmidomycin-added RPMI medium, *SD* standard deviation

## Discussion

Human RBCs have been used to culture blood-stage malaria parasites for the past few decades [25]. More recently, studies employed high-resolution metabolomics to probe host-parasite interaction during blood-stage malaria [6–9, 26, 27]. However, none of the studies probed the variability in RBC metabolites between different independent experiments. Identification of variability in RBC metabolite abundances is critical for making inter-study comparisons and understanding the true impact of parasite infection (or any other perturbation) on RBC metabolism within a study. Here, metabolomic data from five independent experiments were investigated to quantify variability in RBC metabolite abundances. In addition, metabolomic data were also included from a parallel culture of RBCs infected with *P. falciparum* [9] that served as a perturbation standard. It was determined that lipid metabolites (~ 60%) stood out as having ζ values smaller than the median ζ value of all metabolites. This suggests that lipid metabolites tend to change the least between different datasets. In fact, the internal standard metabolite (metabolite with the least variation) identified in this study is also a lipid metabolite (PtdEth C18:0/20:4). The identified internal standard was used to minimize inter-study variations and facilitate dataset comparisons.

Metabolic abundances that vary significantly over time and consistently between the five datasets were the focus of the study. It was determined that the abundance of glycolysis metabolites increased up to two-fold over time. RBCs transport oxygen via haemoglobin, which undergoes autoxidation and produces superoxide radicals [19]. The study’s model simulations revealed that the glycolysis flux primarily enters the PPP (Fig. 5B), the main pathway for producing NADPH. Antioxidant enzymes, such as glutathione peroxidase (GPx), require NADPH to manage low levels of oxidative stress in RBCs [19]. The function of GPx also depends on the availability of glutathione that is synthesized from glutamine or α-ketoglutarate in RBCs [18]. There was an approximate two-fold increase in abundance over time of metabolites (5-oxoproline and α-ketoglutarate) that are involved in glutathione synthesis, suggesting that glutathione synthesis is active in RBCs under all experimental conditions. The model also predicted that GTHO carried substantial flux in the RBC, which also utilizes NADPH to produce glutathione [28], further suggesting active production of glutathione during the experiment.

There was an approximately two-fold increase in the abundance of three dipeptides, namely γ-glutamylisoleucine, γ-glutamylleucine, and γ-glutamylthreonine. RBCs contain an ATP-independent pathway for degrading oxidized haemoglobin [20]. Therefore, the increase in these dipeptides and haem (Table 2) is likely a result of oxidized haemoglobin degradation. The metabolic network analyses of the data also confirmed that glycolysis, PPP, and glutathione metabolism are highly active in the RBCs (Fig. 5A). In addition, the analysis also identified substantial flux through the bicarbonate buffering system. Bicarbonate ions (${\text{HCO}}_{3}^{-}$) are responsible for transporting most of the carbon dioxide with the help of carbonic anhydrase activity [19]. The model suggests that carbonic anhydrase activity is highly variable between different study conditions (Table 3). Since carbonic anhydrase performs reversible hydration of carbon dioxide [29], these results raise the possibility that pH is also variable in the RBCs. Interestingly, the malaria parasites need to maintain a pH that is higher than the host RBCs [30]; thus, the acid load experienced by parasites invading the RBCs would be different for each independent experiment.

Aside from their usage in studying blood-borne pathogens, RBCs are used for transfusion to treat conditions, such as symptomatic anaemia or acute blood loss [31]. Approximately 85 million RBC units are transfused per year [31]. However, RBC transfusion requires storage leading to storage lesions, which may have unintended consequences for the transfusion. The duration of RBC storage, which is between 14 and 21 days at 4 °C, is typically a measure of RBC quality [31]. Although not directly comparable with the storage conditions, it was found that typical consequences of storage lesions, such as haem or oxidative stress, increased two-fold within a two-day experiment at 37 °C. In addition, three dipeptides increased two-fold across all the independent experiments. These results suggest that metabolic markers, such as γ-glutamylleucine and γ-glutamylthreonine, may provide better metrics of RBC quality than storage time for monitoring storage lesions.

### Limitations of the study

By necessity, blood-stage malaria investigators culture *P. falciparum* in human RBCs that originate from different donors in independent studies. In this report, inter-study and time-dependent variability in abundances of robustly detected (> 1000 raw counts) RBC metabolites were characterized. Metabolomic data obtained under in vitro culture conditions, containing nutrient-rich medium (RPMI) and a lipid supplement (AlbuMAX), were used. This culture medium composition facilitates in vitro* P. falciparum* growth, but may also contribute to the time-dependent variation in RBC metabolite abundances (Table 2) because the in vivo RBC environment in the bloodstream is not similar to these in vitro conditions. Regardless of the medium used, the RBC metabolism of the donor would always contribute to time-dependent adjustments in RBC metabolism due to the in vitro incubation. The RPMI-based medium was chosen since it is used for essentially all experiments with cultured *P. falciparum* parasites. To mitigate inter-study variability associated with donor metabolism, an internal standard metabolite was identified to normalize raw metabolomic data from the five independent studies. The normalization method proposed in this study would be useful for making any inter-study comparisons; however, the internal standard metabolite may (or may not) be PtdEth (C18:0/20:4) because of the limited number of independent datasets (N = 5) analysed in this work.

## Conclusions

High-resolution metabolomic data were analysed from five independent experiments, maintaining uninfected RBCs under near-identical culture conditions for two days and using the identical experimental protocols originally designed for *P. falciparum* blood-stage studies. These culture systems provide the ex vivo human host background environment for laboratory studies of *P. falciparum* and other blood-borne pathogens. To facilitate comparison between metabolites in different studies, an internal standard metabolite was identified (PtdEth C18:0/20:4) that had minimal inter-study and time-dependent variability. Based on normalization with this standard, approximately 200 metabolites were selected that were robustly (metabolite raw count > 1000) detected across all replicates at all sampled time points for the five independent experiments and determined their intrinsic variability in this culture system. This set provides a broadly applicable standard variability benchmark for the most commonly detectable metabolites. Overall, alterations in metabolite abundances over time were consistent between independent experiments. Time-dependent metabolite changes were mainly detected for metabolic processes related to RBC glycolysis and stress responses that occur over the course of the 48-h culture time period. A smaller set of metabolites that vary substantially in uninfected RBCs between the different studies was also identified; these changes reflect non-specific metabolite variability due to unknown factors, such as differences in the metabolic status of blood donors. This latter set of metabolites can be useful in identifying culture conditions that affect RBC metabolism itself and, thus, potentially obscuring changes caused by blood-borne pathogens.

## Supplementary Information

**Additional file 1: **Raw metabolomic data obtained in quadruplicate from uRBC cultures at 0, 8, 16, 24, 32, and 40 hours (Sheet 1). Raw metabolomic data collected in the four published studies (Sheets 2–5).

**Additional file 2: Text S1.** detailing the data integration technique used for predicting RBC metabolism. **Figure S1.** showing probability distribution of the estimated ζ values; and **Figures S2–S30** illustrating RBC metabolic states at 8, 16, 24, 32, and 40 hours during the “Pure 1” study and at every sampled time point of the other four studies.

**Additional file 3. **List of overlapping metabolites with significant raw count (>1000) and their respective ζ values.

**Additional file 4: **Maximum and minimum flux of metabolic enzymes listed in Table 3 during the five studies.

**Additional file 5: **Absolute differences of five high-magnitude fluxes within uRBCs of the five studies compared with their estimated fluxes in iRBCs.

**Additional file 6: **Absolute differences of substantially varying fluxes, listed in Table 3, within uRBCs of the five studies compared with their estimated fluxes in iRBCs.

## Data Availability

All data generated or analysed during this study are included in this published article and its additional files.

## Abbreviations

+ Fos: Fosmidomycin-added RPMI medium
+ Mev: Mevalonate-added RPMI medium
ATP: Adenosine triphosphate
DGPase: Diphosphoglycerate phosphatase
DPGM: Diphosphoglyceromutase
ENO: Enolase
FBA: Fructose biphosphate aldolase
GAPD: Glyceraldehyde 3-phosphate dehydrogenase
gDW: Gram dry weight
glc-D(e): Medium d-glucose
GLCt1r: Glucose transporter
GPx: Glutathione peroxidase
GTHO: Glutathione oxidoreductase
HCA: Hierarchical clustering analysis
HEX1: Hexokinase
-Hxn: Hypoxanthine-limited RPMI medium
WHO: World Health Organization
