# Supplementary material for: Inter-study and time-dependent variability of metabolite abundance in cultured red blood cells
Source: Malar J. 2021 Jul 2;20:299. doi: 10.1186/s12936-021-03780-5 (PMC8254254; doi:10.1186/s12936-021-03780-5)
Supplement: Supplementary file 2 — Additional file 2: Text S1. detailing the data integration technique used for predicting RBC metabolism. Figure S1. showing probability distribution of the estimated ζ values; and Figures S2–S30 illustrating RBC metabolic states at 8, 16, 24, 32, and 40 hours during the “Pure 1” study and at every sampled time point of the other four studies. [file 12936_2021_3780_MOESM2_ESM.pdf]

Electronic Supplementary Material for the paper (Additional file 2):

Inter-study and time-dependent variability of metabolite abundance in cultured red blood cells

By

Shivendra G. Tewari*, Krithika Rajaram, Russell P. Swift, Bobby Kwan, Jaques Reifman, Sean T. Prigge, and Anders Wallqvist*

***Corresponding author. Email(s): stewari@bhsai.org or sven.a.wallqvist.civ@mail.mil

This PDF file includes: Text S1 and Figures S1–S30.

Figure S1: Probability distribution of ζ values of all overlapping metabolites. We used the mean and standard deviation of the ζ values to compute the z-values (x-axis). The number on top of each bar indicates the number of metabolites within the interval. The internal standard metabolite, phosphatidylethanolamine (PtdEth) C18:0/20:4, was the least varying metabolite based on the estimated probabilities ($p < 0.001$).

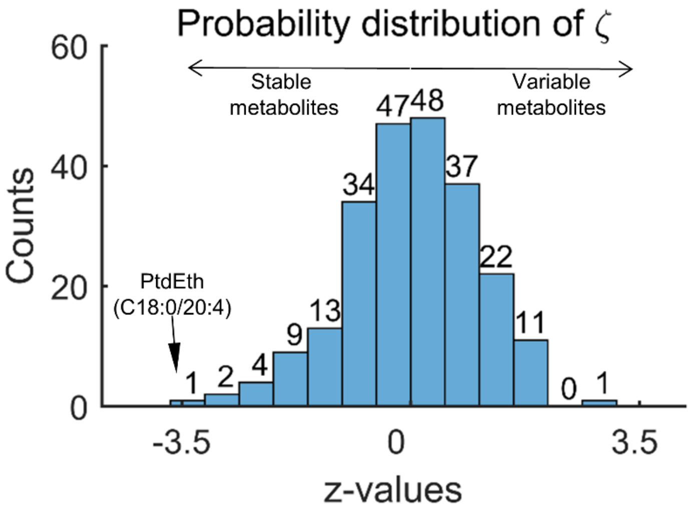

Text S1. Integrating metabolomic data with red blood cell metabolic network

Estimating nutrient uptake and secretion rates of metabolite in the culture medium

We first quantified the net uptake and secretion rates of nutrients in the red blood cells (RBCs) maintained in the Roswell Park Memorial Institute (RPMI) medium. Towards this end, we used the extracellular metabolomic data published by Olszweski et al. (1) to calculate average and time-sensitive nutrient uptake (or secretion) rates in the RBCs. First, we calculated uptake/secretion rates at each sampled time point, denoted by v_{nut}^{t}, via linear regression, by fitting relative levels of culture medium metabolites (weighted by their standard deviation) as a function of time for [*t*, *t*+2] points, where *t* is the time point for which the rate is being calculated. Using the initial concentration of the metabolite and the dry weight (DW) of an erythrocyte, we converted the estimated slopes of the regression lines (uptake/secretion rates) to units of mmol $hr^{-1}gDW^{-1}$. To identify average nutrient uptake (or secretion), we followed the same procedure but fitted the relative levels of each metabolite at all sampled time points, i.e., 0, 8, 16, 24, 32, and 40 h. Although Olszewski et al. (1) measured 59 culture medium metabolites, we were only able to compute uptake (or secretion) rates of 22 metabolites, due to unavailability of initial concentration, listed below in Table S1.

Table S1: Estimated nutrient uptake or secretion rates in RBC*

Metabolite	Mean	95% Confidence interval
Arginine	-2.8×10^{-3}	$[-2.8\times10^{-2}, 2.3\times10^{-2}]$
Asparagine	1.0×10^{-2}	$[-2.8\times10^{-4}, 2.1\times10^{-2}]$
Aspartate	-1.1×10^{-3}	$[-3.7\times10^{-3}, 1.5\times10^{-3}]$
Choline	9.3×10^{-4}	$[6.5\times10^{-4}, 1.2\times10^{-3}]$
Glucose	-2.9×10^{-1}	$[-4.2\times10^{-1}, -1.6\times10^{-1}]$
Glutamate	4.9×10^{-3}	$[3.1\times10^{-5}, 9.8\times10^{-3}]$
Glutamine	1.1×10^{-1}	$[-1.3\times10^{-3}, 2.1\times10^{-1}]$
Histidine	1.8×10^{-3}	$[-1.2\times10^{-3}, 4.7\times10^{-3}]$
Hypoxanthine†	-1.9×10^{-3}	$[-2.9\times10^{-3}, -8.1\times10^{-4}]$
Methionine	1.6×10^{-2}	$[-7.9\times10^{-3}, 2.4\times10^{-2}]$
Inositol	-7.7×10^{-3}	$[-1.1\times10^{-2}, -3.8\times10^{-3}]$
Nicotinamide	-1.2×10^{-6}	$[-2.4\times10^{-4}, 2.4\times10^{-4}]$
Pantothenate	-2.2×10^{-5}	$[-3.2\times10^{-5}, -1.2\times10^{-5}]$
Phenylalanine	2.1×10^{-2}	$[1.2\times10^{-2}, 3.0\times10^{-2}]$
Proline	2.3×10^{-3}	$[-2.0\times10^{-3}, 6.6\times10^{-3}]$
Pyridoxine	-2.4×10^{-4}	$[-3.5\times10^{-4}, -1.3\times10^{-4}]$
Riboflavin	3.9×10^{-5}	$[6.1\times10^{-6}, 7.3\times10^{-5}]$
Serine	2.2×10^{-4}	$[-1.0\times10^{-2}, 1.0\times10^{-2}]$
Thiamin	2.0×10^{-4}	$[1.0\times10^{-4}, 2.9\times10^{-4}]$
Tryptophan	6.6×10^{-3}	$[4.1\times10^{-3}, 9.1\times10^{-3}]$
Tyrosine	7.4×10^{-3}	$[3.2\times10^{-3}, 1.2\times10^{-2}]$
Valine	2.8×10^{-2}	$[1.8\times10^{-2}, 3.9\times10^{-2}]$

*All rates are in units of mmol/h/gDW. The negative values indicate uptake, while positive values indicate secretion of a metabolite. †The estimated uptake value of hypoxanthine in the hypoxanthine-limited RPMI medium (2) was -1.9×10^{-5} $[-2.9\times10^{-3}, -8.1\times10^{-4}]$.

Using metabolomic data to estimate RBC metabolism

Prior to integrating the intracellular metabolomic data with the red blood cell (RBC) network model, we first identified directionality of RBC metabolic reactions by performing a flux variability analysis using "fluxVariability" function built-in to the COnstraint-Based Reconstruction and Analysis (COBRA) toolbox (3), while not allowing any loops in the final solution, and keeping the sodium pump activity of RBC at 1 mmol/h/gDW, denoted by μ below. The chosen sodium pump activity is 35% of its theoretical maximum in the model and roughly corresponds to the sodium pump activity in packed red blood cells (4). By performing this flux variability step, we identified a set of unidirectional reactions R, used later for integrating the metabolomic data with the model.

We used the average nutrient uptake and secretion rates, listed in Table S1, to identify the basal metabolism (v_{bas}) of RBC in the RPMI medium. Mathematically, we solved the following equation:

$$\max|v_{NaK}| \quad (1)$$

$$\text{subject to: } S_{RBC}\cdot \mathrm{v} = 0,\ \sum_{j\in N}\left|v_j - v_j^{est}\right| < \varepsilon, \text{ and } \mathrm{v}_{lb} \le \mathrm{v} \le \mathrm{v}_{ub}.$$

Here, S_{RBC} is a matrix containing the stoichiometry of RBC metabolic reactions; v represents a vector containing metabolic reactions in the RBC; *N* denotes the set of reactions associated with nutrient uptake/secretion; v_j^{est} is the estimated uptake (or secretion) rate of jth nutrient, listed in Table S1; v_{lb} and v_{ub} are vectors representing the lower and upper bounds, respectively, of metabolic reactions in the RBC; ε denotes the minimal value of $\left|v_j - v_j^{est}\right|$ subjected to the same stoichiometric and boundary constraints, and basal sodium pump activity μ.

After calculating the basal RBC metabolism (v_{bas}), we integrated time-resolved metabolomic data with the RBC network to predict medium-specific RBC metabolism. Towards this end, we first solved the following equation:

$$\delta^t = \min\left[\sum_{i\in R} v_i^t - \alpha_i^t \cdot v_{i,bas} + \sum_{j\in N} v_j^t - v_{j,nut}^t]\right] \quad (2)$$

$$\text{subject to: } S_{RBC}\cdot \mathrm{v} = 0 \text{ and } \mathrm{v}_{lb} \le \mathrm{v} \le \mathrm{v}_{ub}$$

$$\min\sum_{i\in M}\left|v_i^t - v_{i,bas}\right| \quad (3)$$

$$\text{subject to: } \sum_{i\in R} v_i^t - \alpha_i^t \cdot v_{i,bas} + \sum_{j\in N} v_j^t - v_{j,nut}^t \le \delta^t$$

$$S_{RBC}\cdot \mathrm{v} = 0 \text{ and } \mathrm{v}_{lb} \le \mathrm{v} \le \mathrm{v}_{ub}$$

Here, α^t is a column vector with each row-element containing the minimal value of all the metabolites taking part in the row reaction at a given time t; before taking the minimal value of the participating metabolites, each metabolite is normalized by its median value during the experiment. R denotes a set of unidirectional reactions determined using the flux variability step (discussed above), and M represents the set of all metabolic reactions in the RBC. $v_{i,bas}$ and $v_{j,bas}$ denote the ith and j^{th} values, respectively, of the column vector v_{bas}. $v_{j,nut}^t$ represents the jth value of the column vector v_{nut}^t. We followed these steps for calculating RBC metabolism under Pure 1, -Hxn, +Mev, +Fos, and Pure 2 medium conditions. Figures S2–S30 illustrate the metabolic state of RBC under these conditions at each sampled time point.

Figure S2: Overview of estimated metabolic fluxes at 8 h in red blood cells (RBC) maintained under ‘Pure 1’ medium condition.

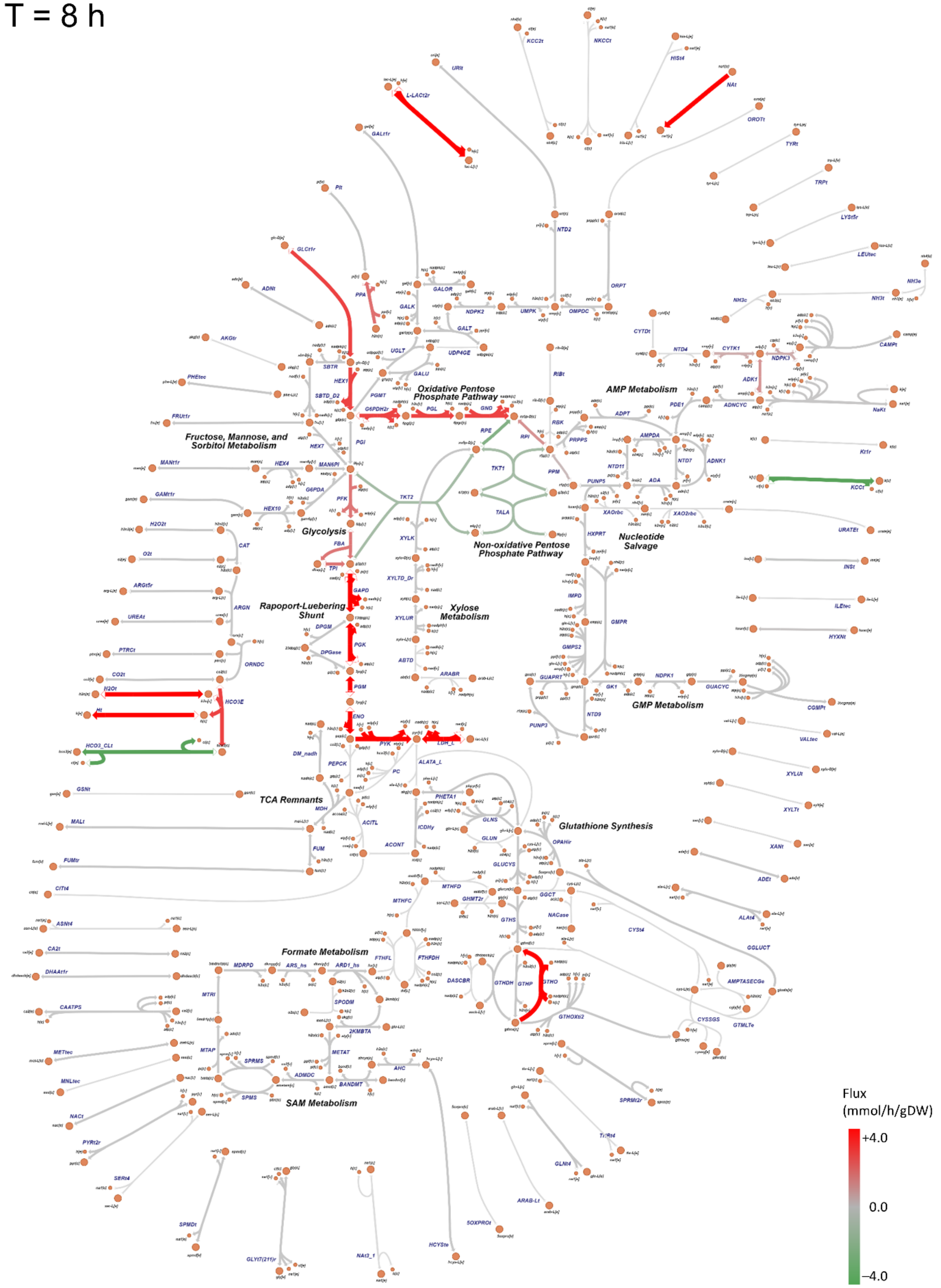

Figure S3: Overview of estimated metabolic fluxes at 16 h in red blood cells (RBC) maintained under ‘Pure 1’ medium condition.

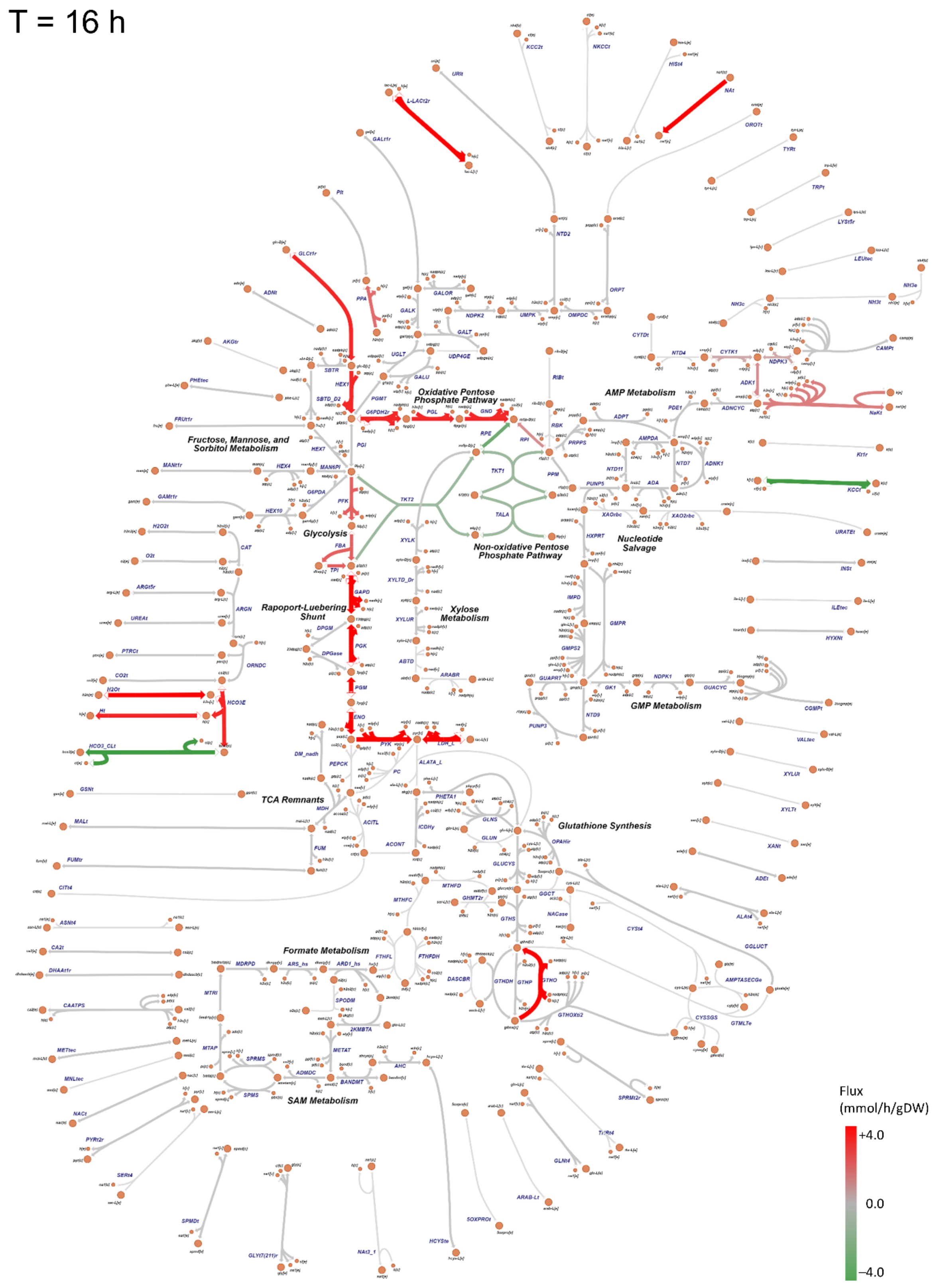

Figure S4: Overview of estimated metabolic fluxes at 24 h in red blood cells (RBC) maintained under ‘Pure 1’ medium condition.

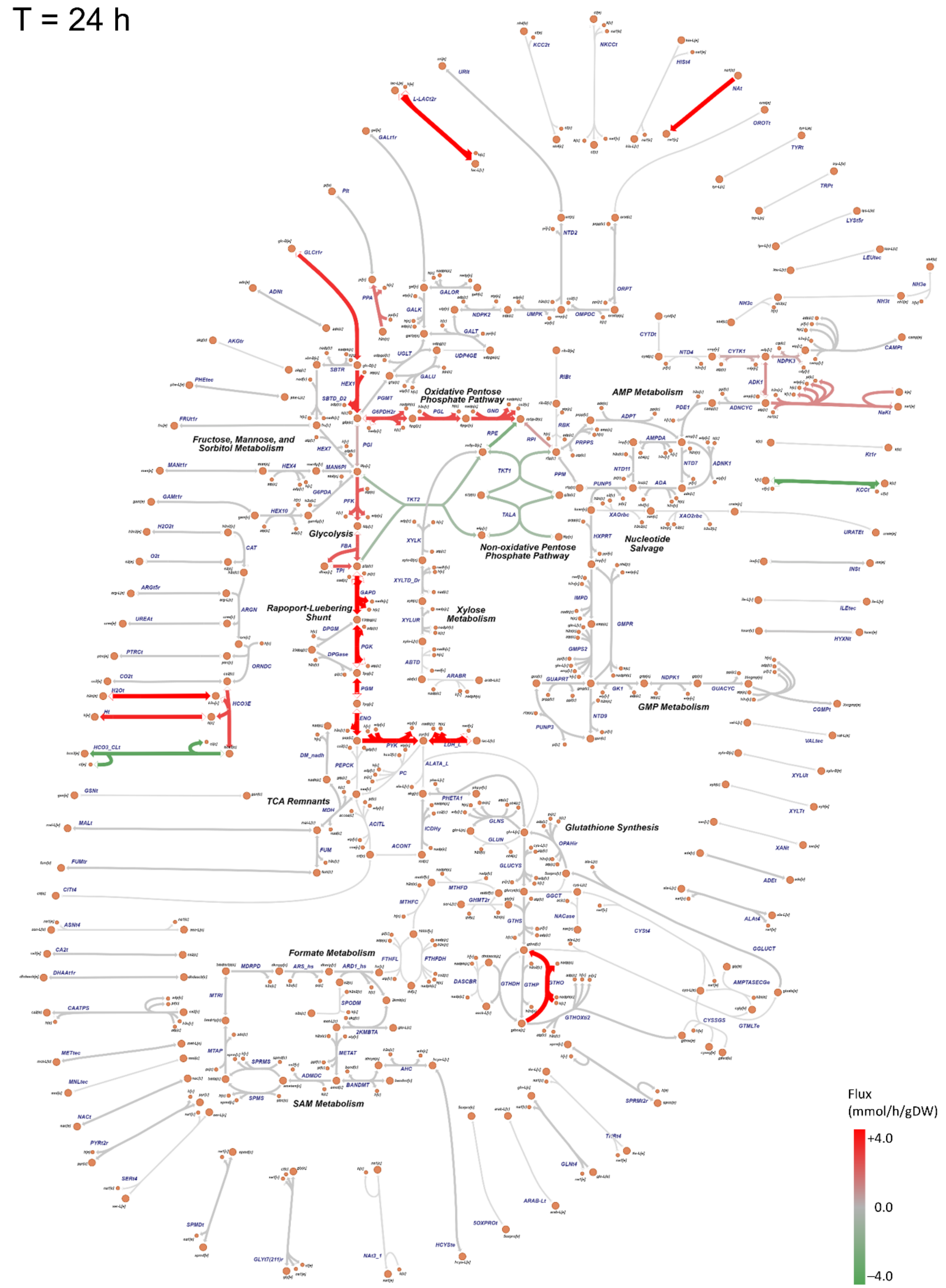

Figure S5: Overview of estimated metabolic fluxes at 32 h in red blood cells (RBC) maintained under ‘Pure 1’ medium condition.

T = 32 h

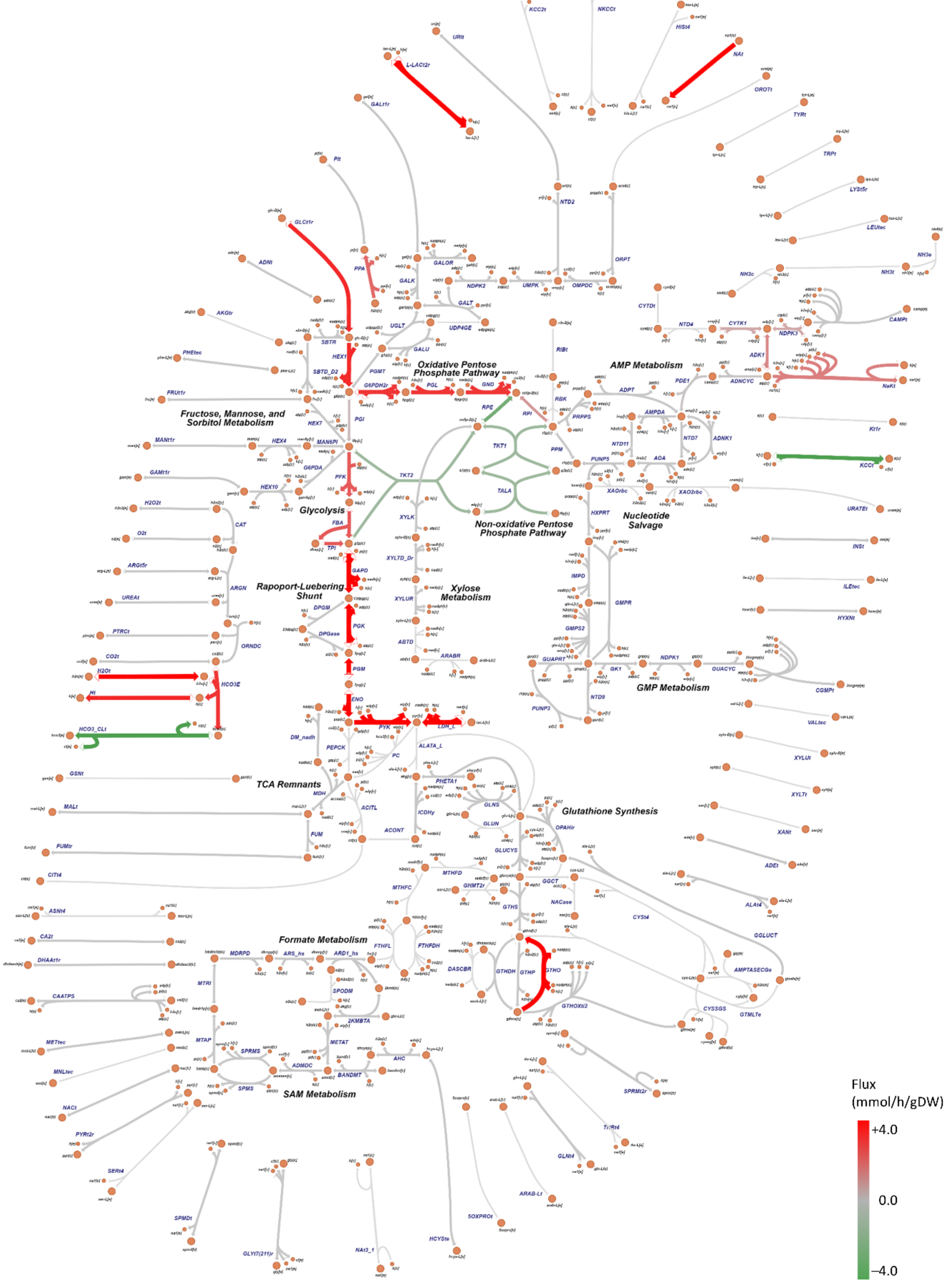

Figure S6: Overview of estimated metabolic fluxes at 40 h in red blood cells (RBC) maintained under ‘Pure 1’ medium condition.

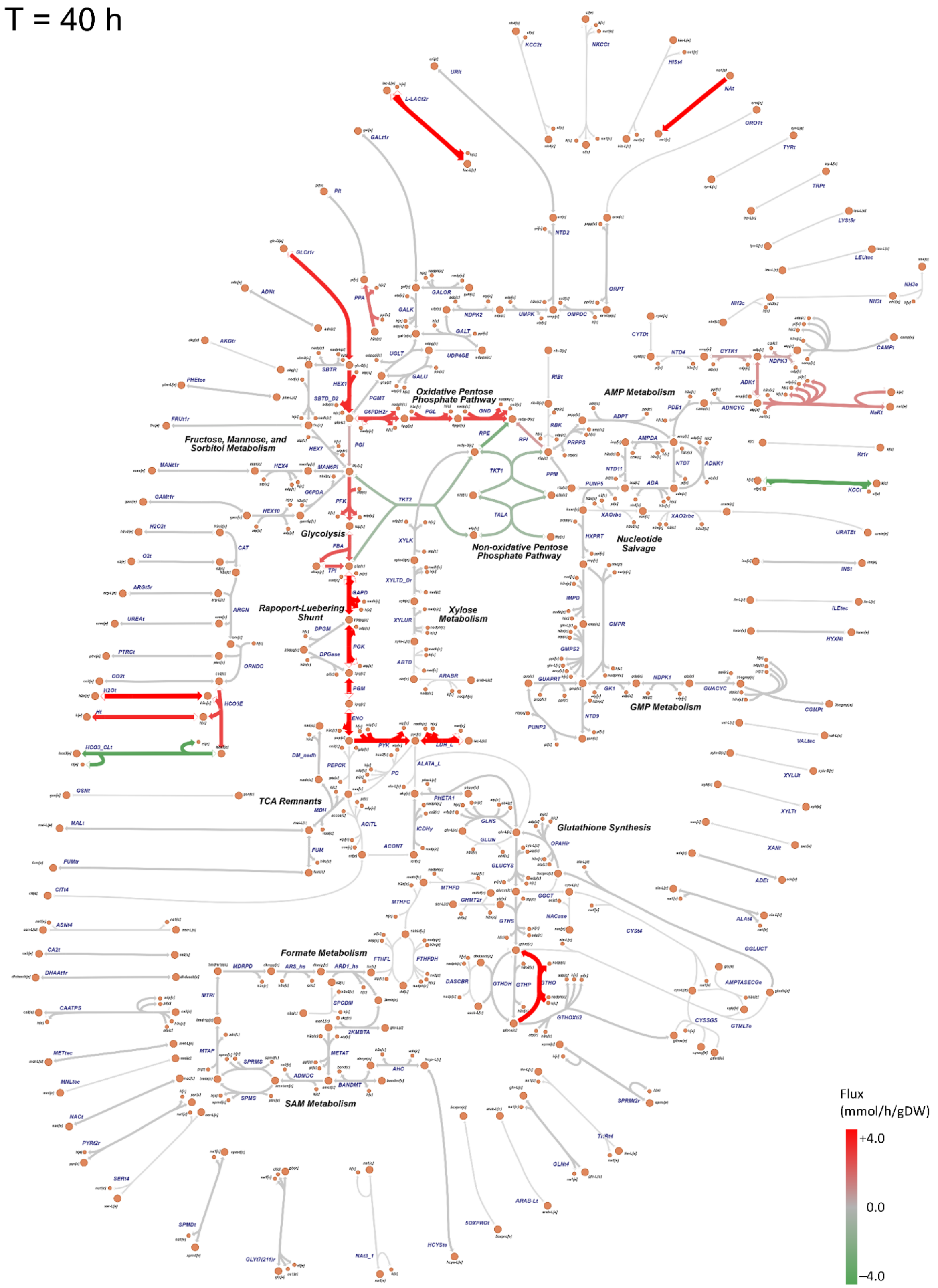

Figure S7: Overview of estimated metabolic fluxes at 0 h in red blood cells (RBC) maintained under '-Hxn' medium condition.

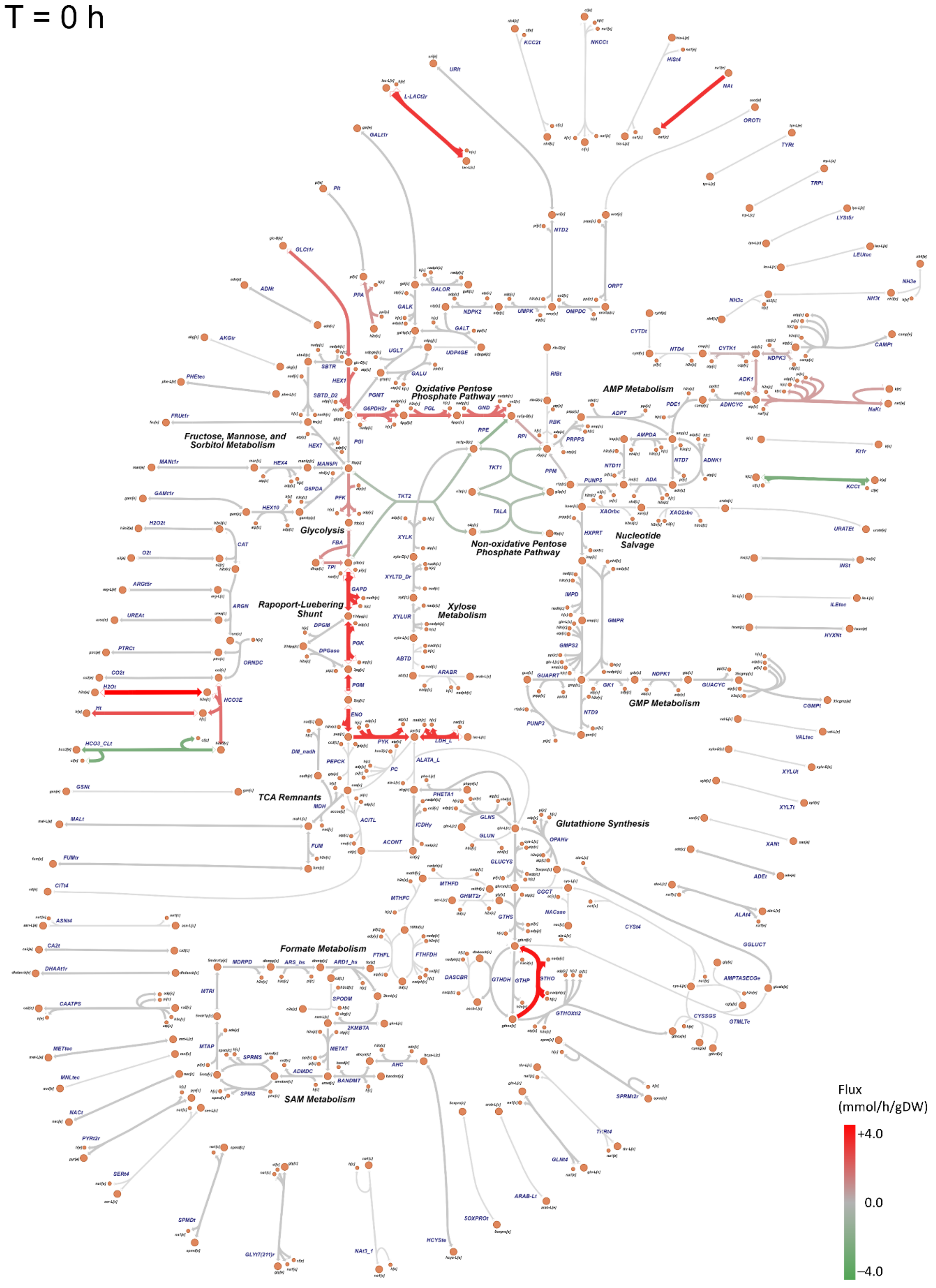

Figure S8: Overview of estimated metabolic fluxes at 8 h in red blood cells (RBC) maintained under '-Hxn' medium condition.

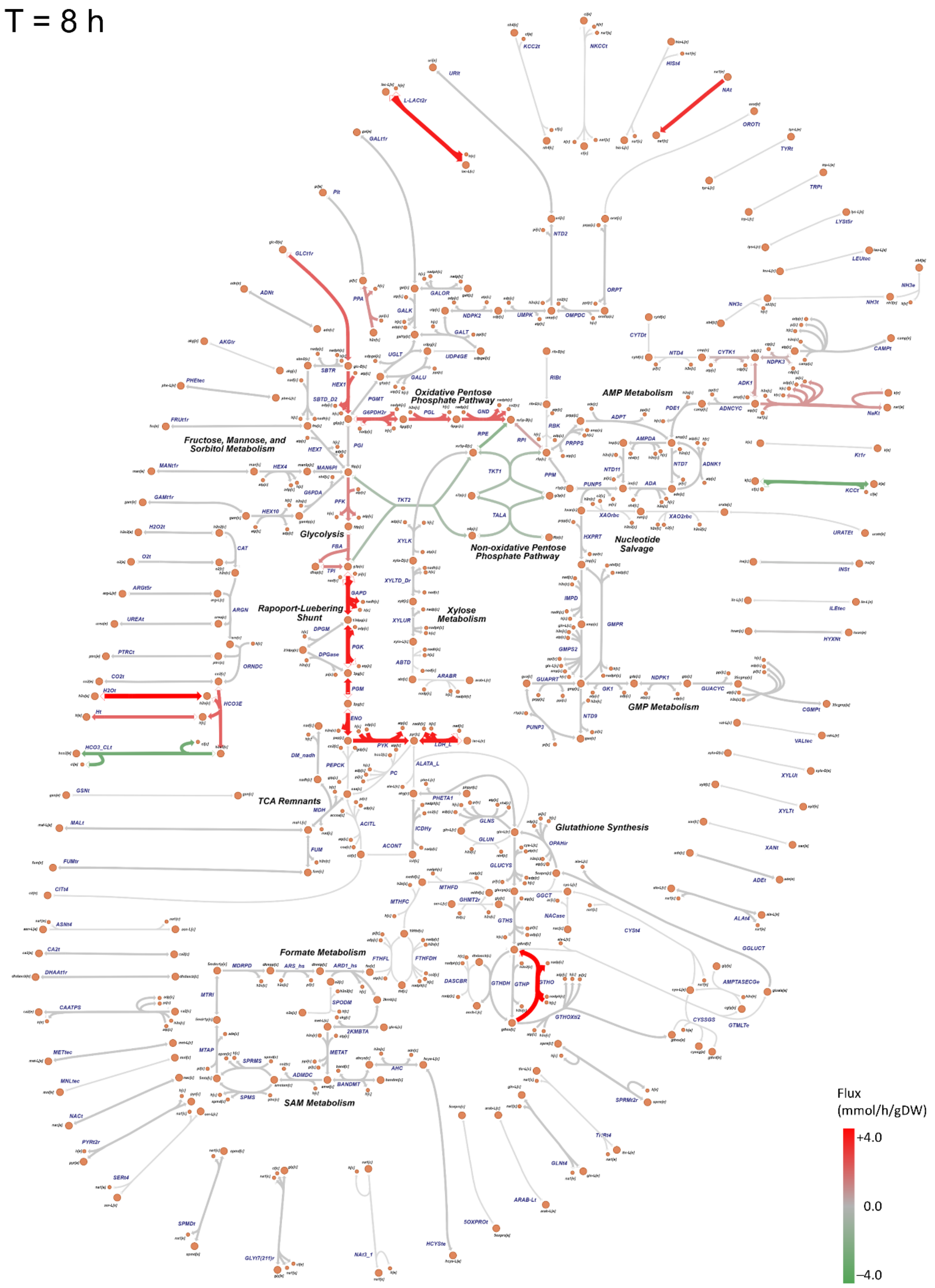

Figure S9: Overview of estimated metabolic fluxes at 16 h in red blood cells (RBC) maintained under ‘-Hxn’ medium condition.

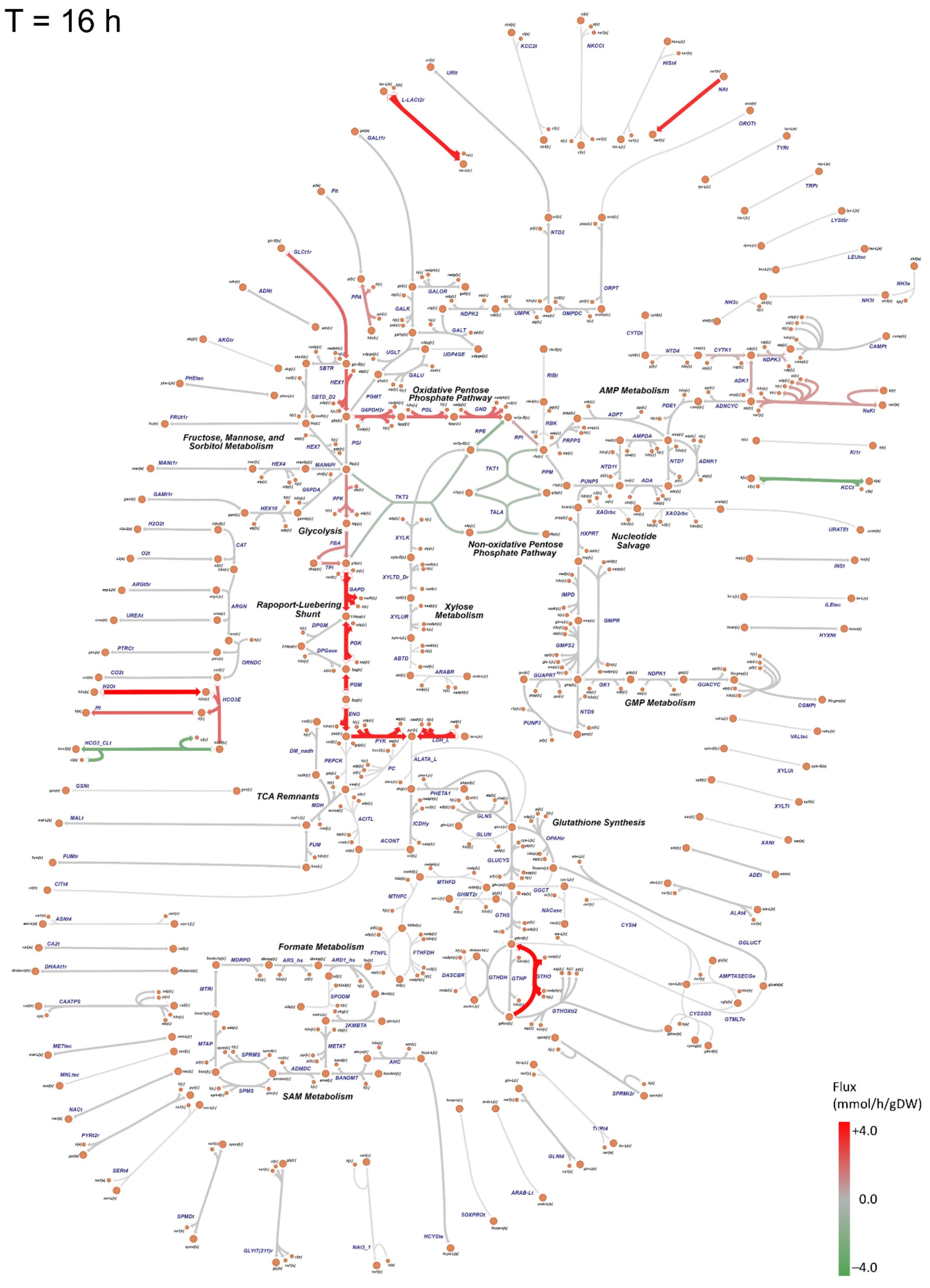

Figure S10: Overview of estimated metabolic fluxes at 24 h in red blood cells (RBC) maintained under '-Hxn' medium condition.

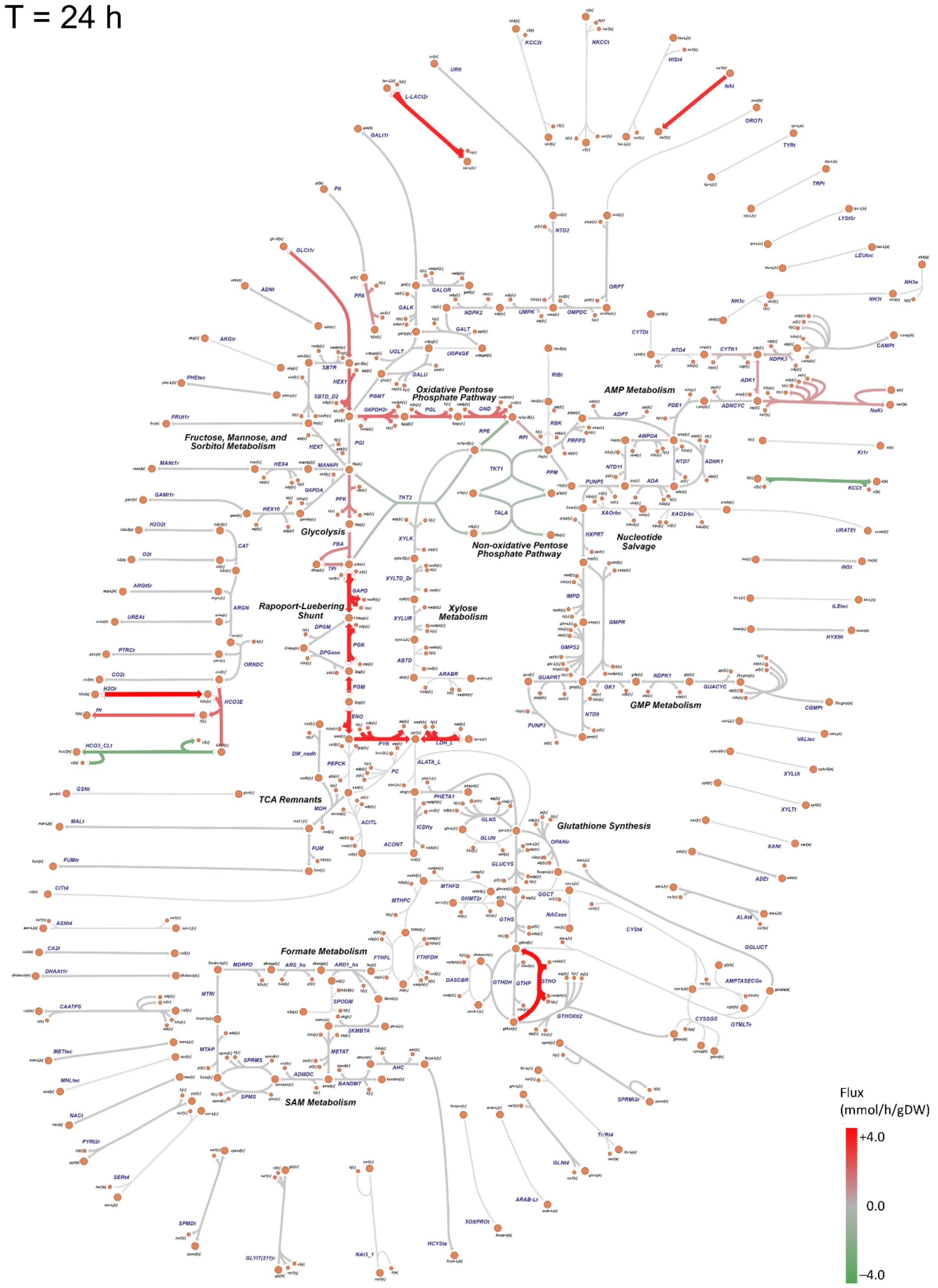

Figure S11: Overview of estimated metabolic fluxes at 32 h in red blood cells (RBC) maintained under '-Hxn' medium condition.

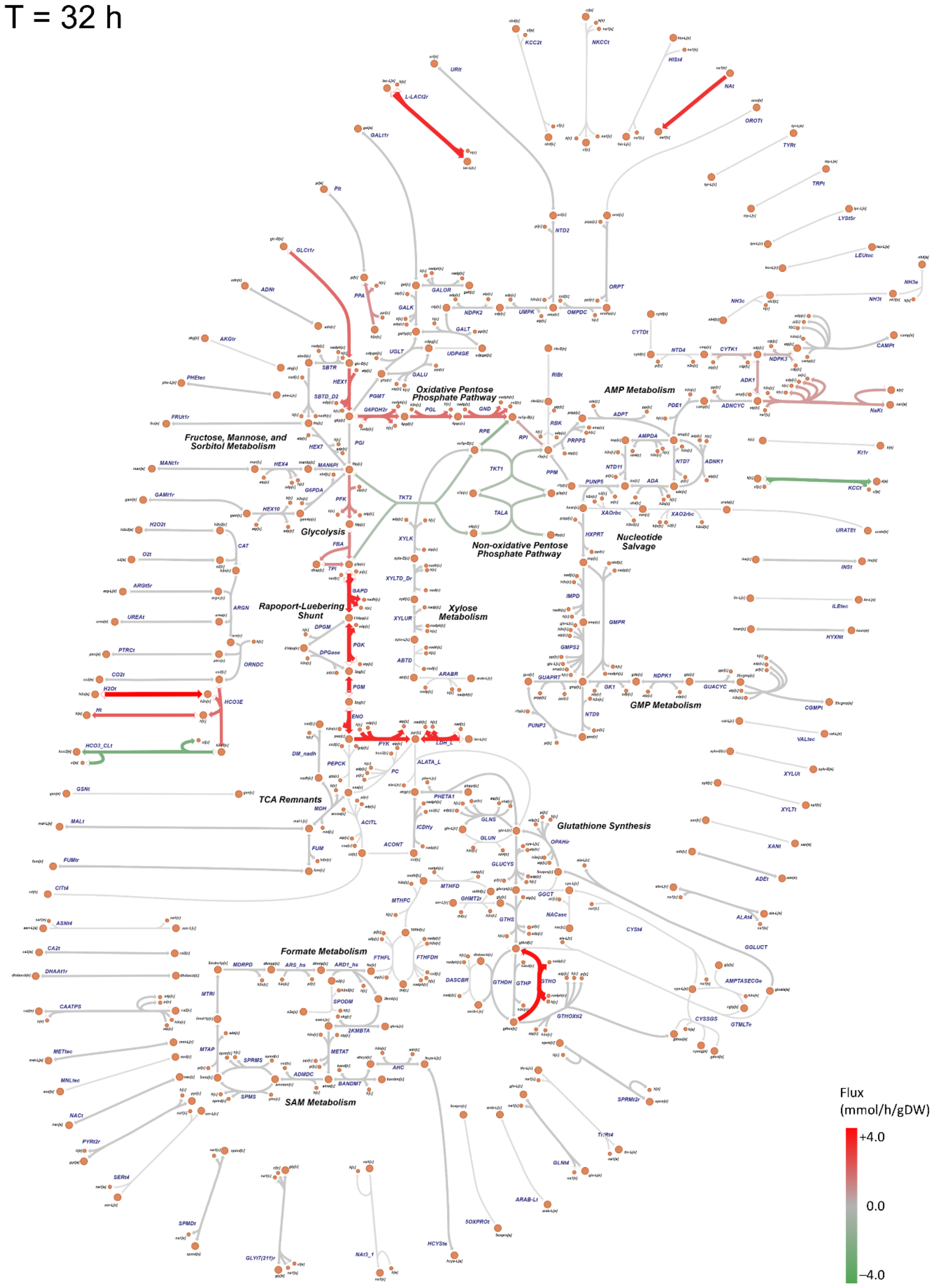

Figure S12: Overview of estimated metabolic fluxes at 40 h in red blood cells (RBC) maintained under '-Hxn' medium condition.

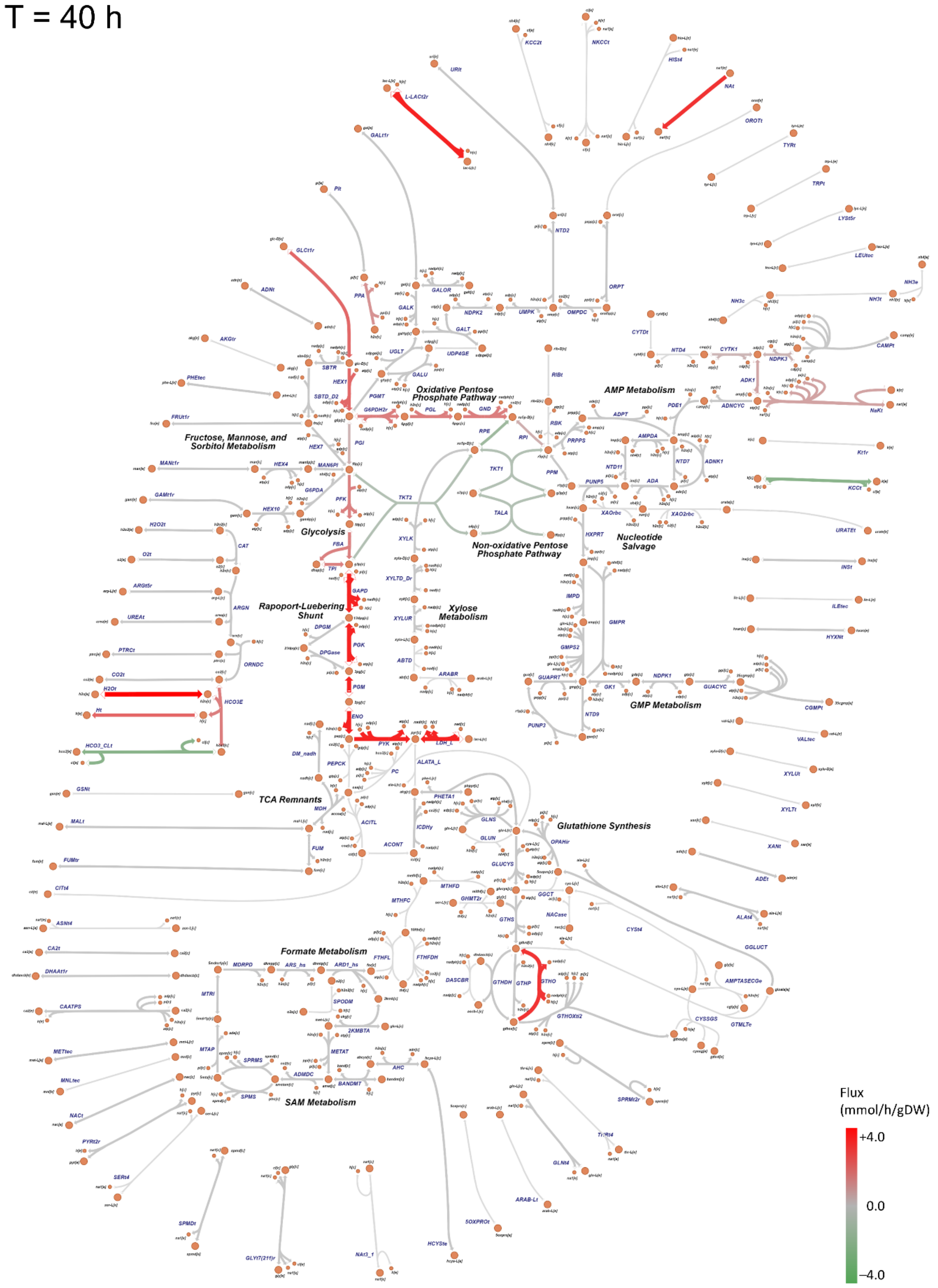

Figure S13: Overview of estimated metabolic fluxes at 0 h in red blood cells (RBC) maintained under ‘+Mev’ medium condition.

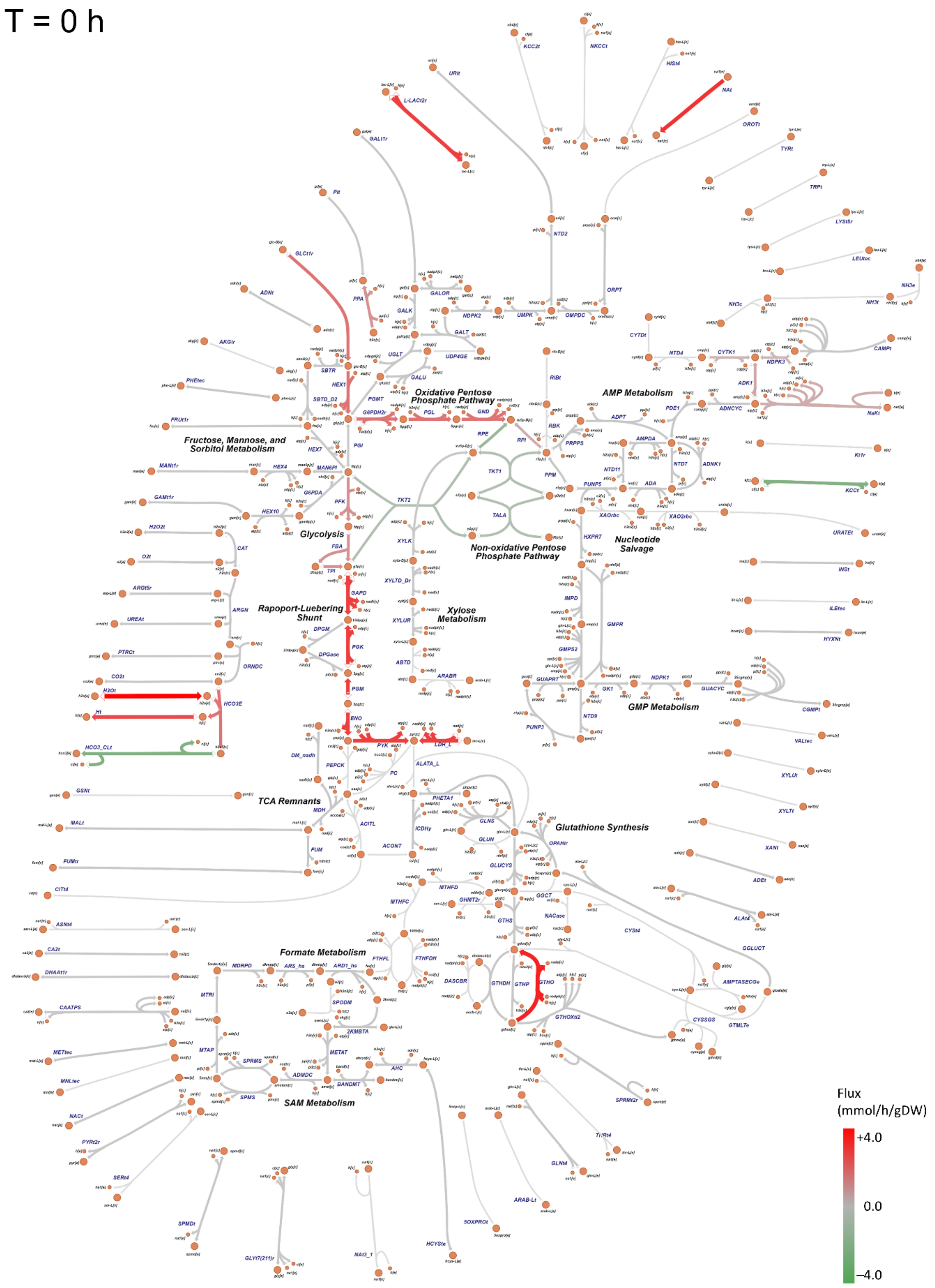

Figure S14: Overview of estimated metabolic fluxes at 8 h in red blood cells (RBC) maintained under '+Mev' medium condition.

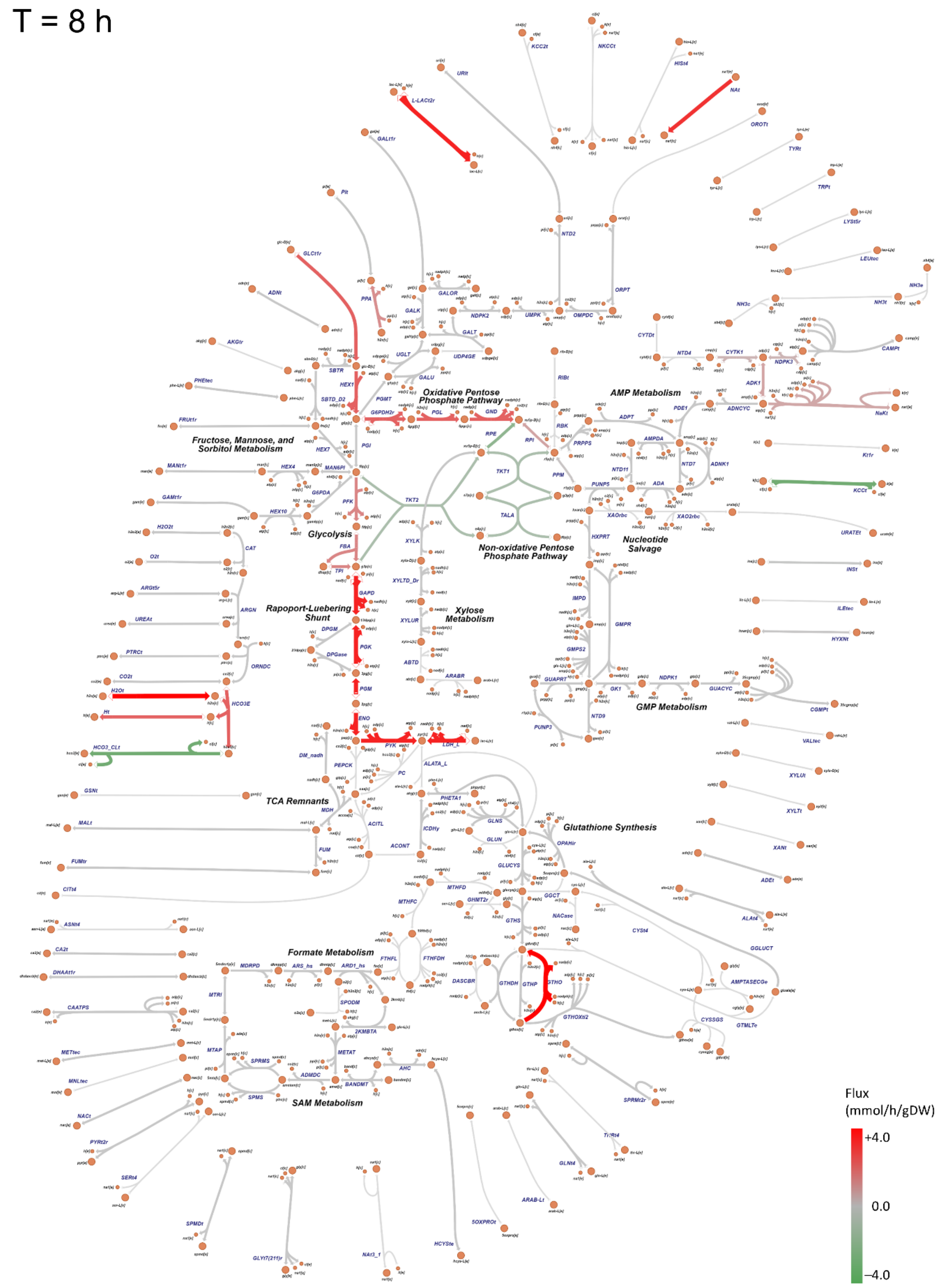

Figure S15: Overview of estimated metabolic fluxes at 16 h in red blood cells (RBC) maintained under '+Mev' medium condition.

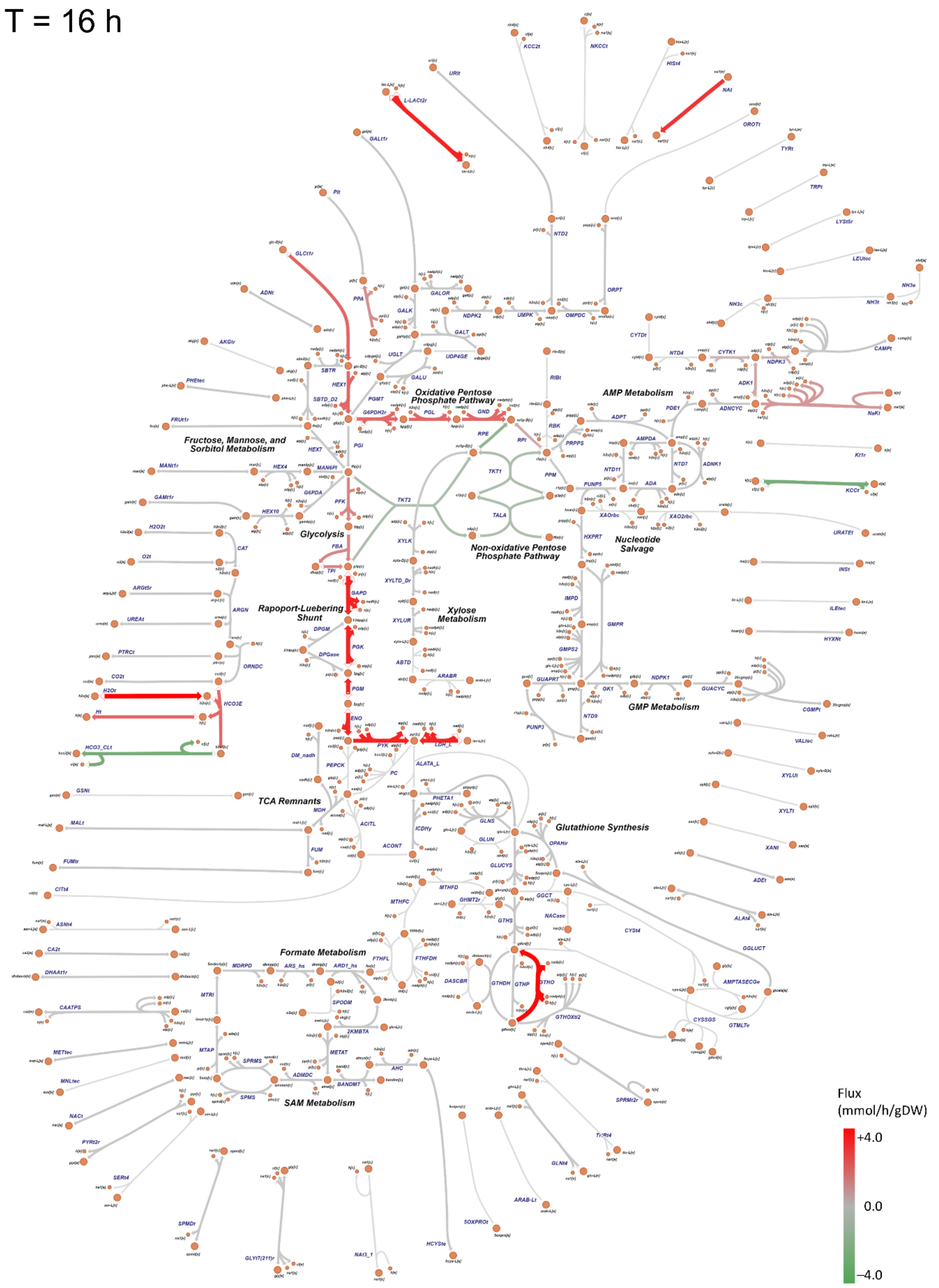

Figure S16: Overview of estimated metabolic fluxes at 24 h in red blood cells (RBC) maintained under ‘+Mev’ medium condition.

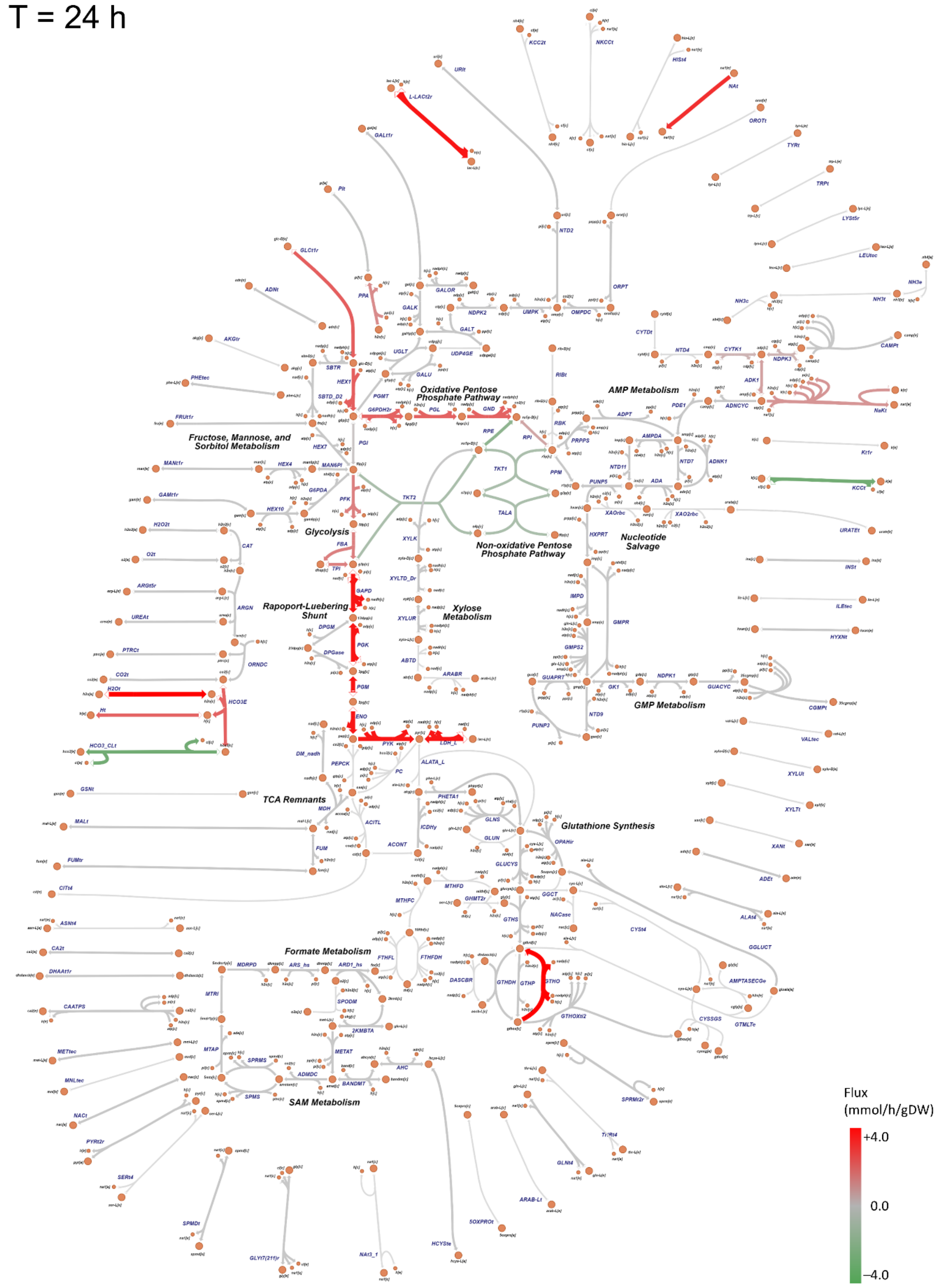

Figure S17: Overview of estimated metabolic fluxes at 32 h in red blood cells (RBC) maintained under ‘+Mev’ medium condition.

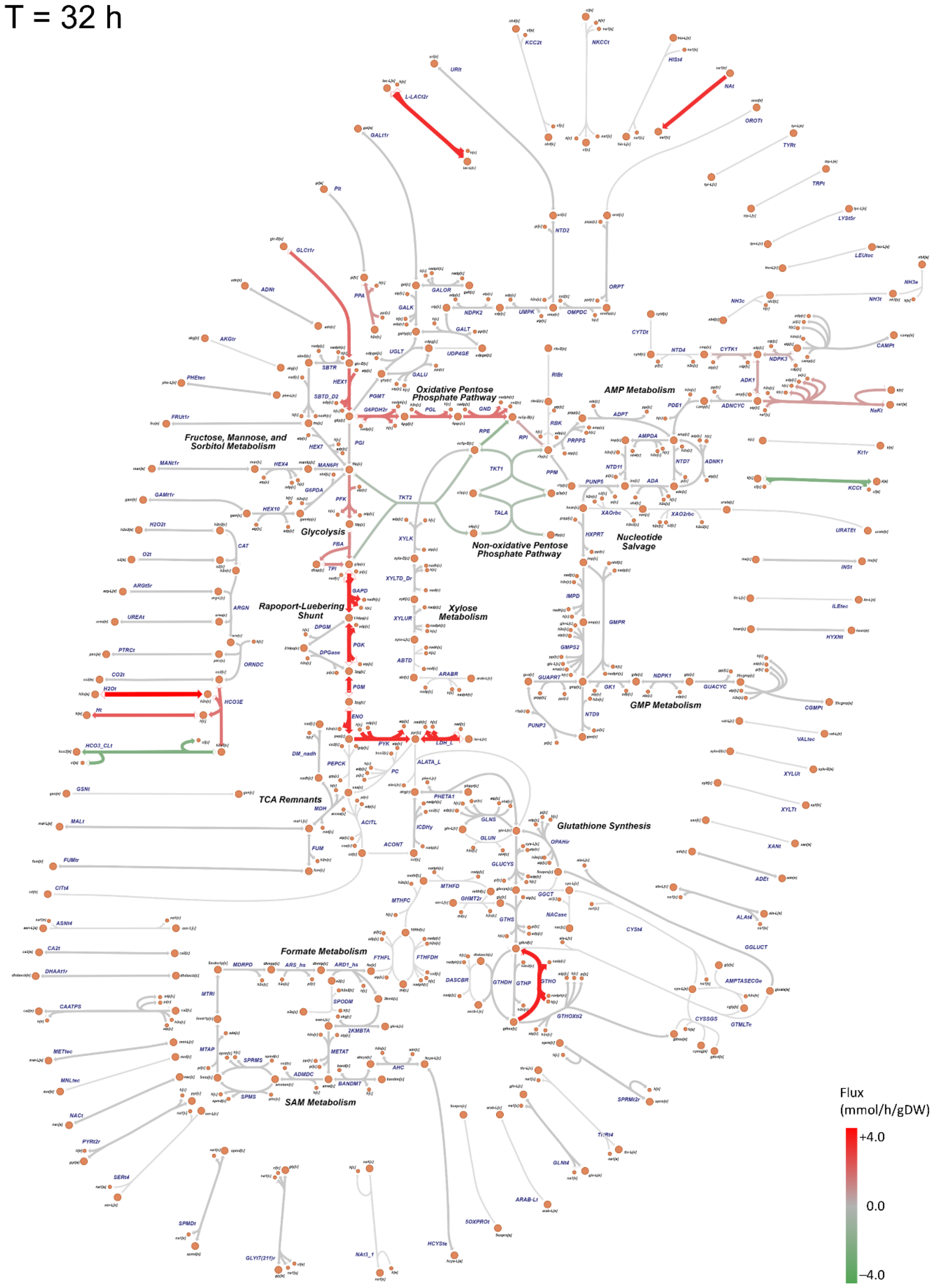

Figure S18: Overview of estimated metabolic fluxes at 40 h in red blood cells (RBC) maintained under ‘+Mev’ medium condition.

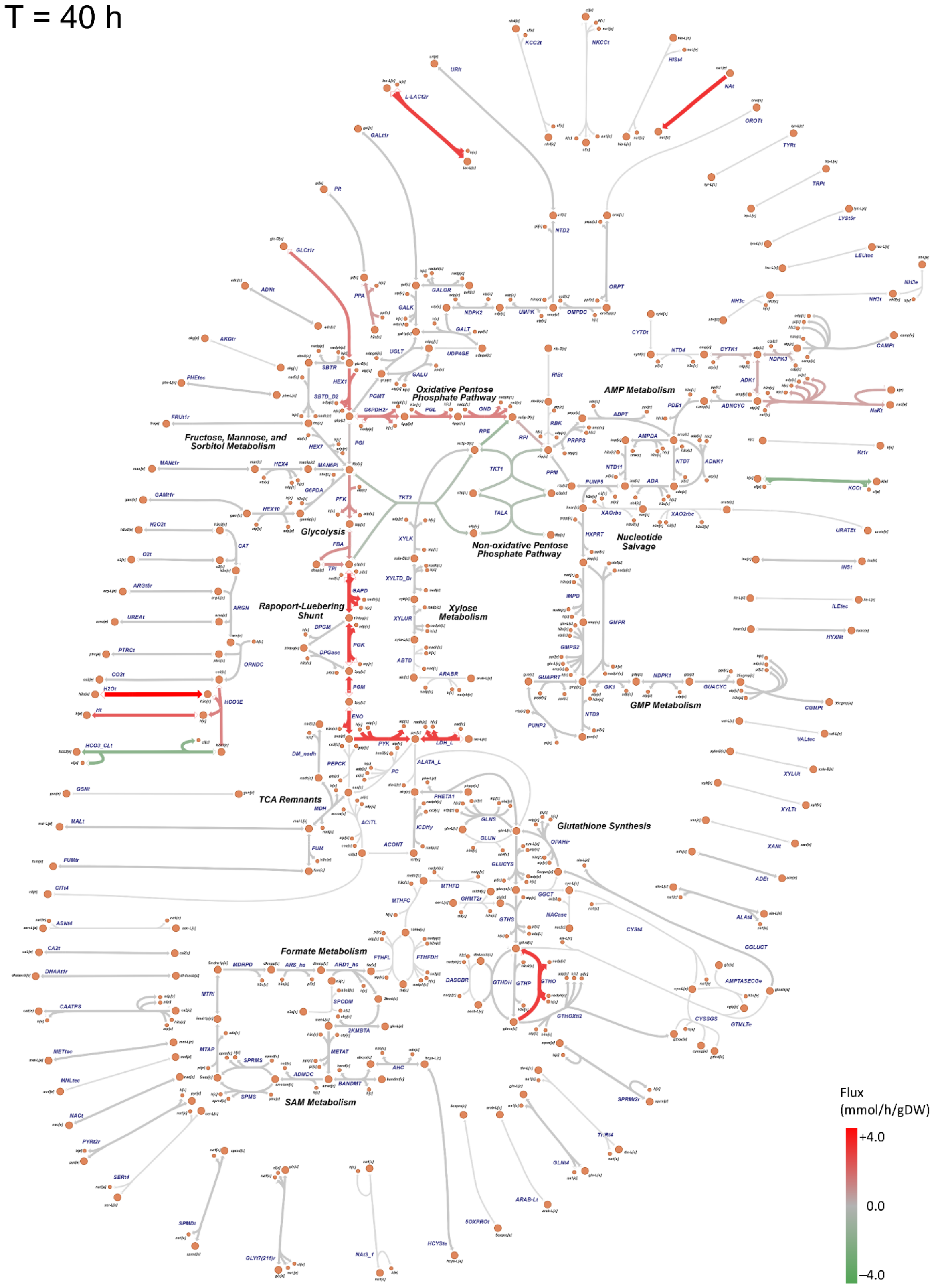

Figure S19: Overview of estimated metabolic fluxes at 0 h in red blood cells (RBC) maintained under '+Fos' medium condition.

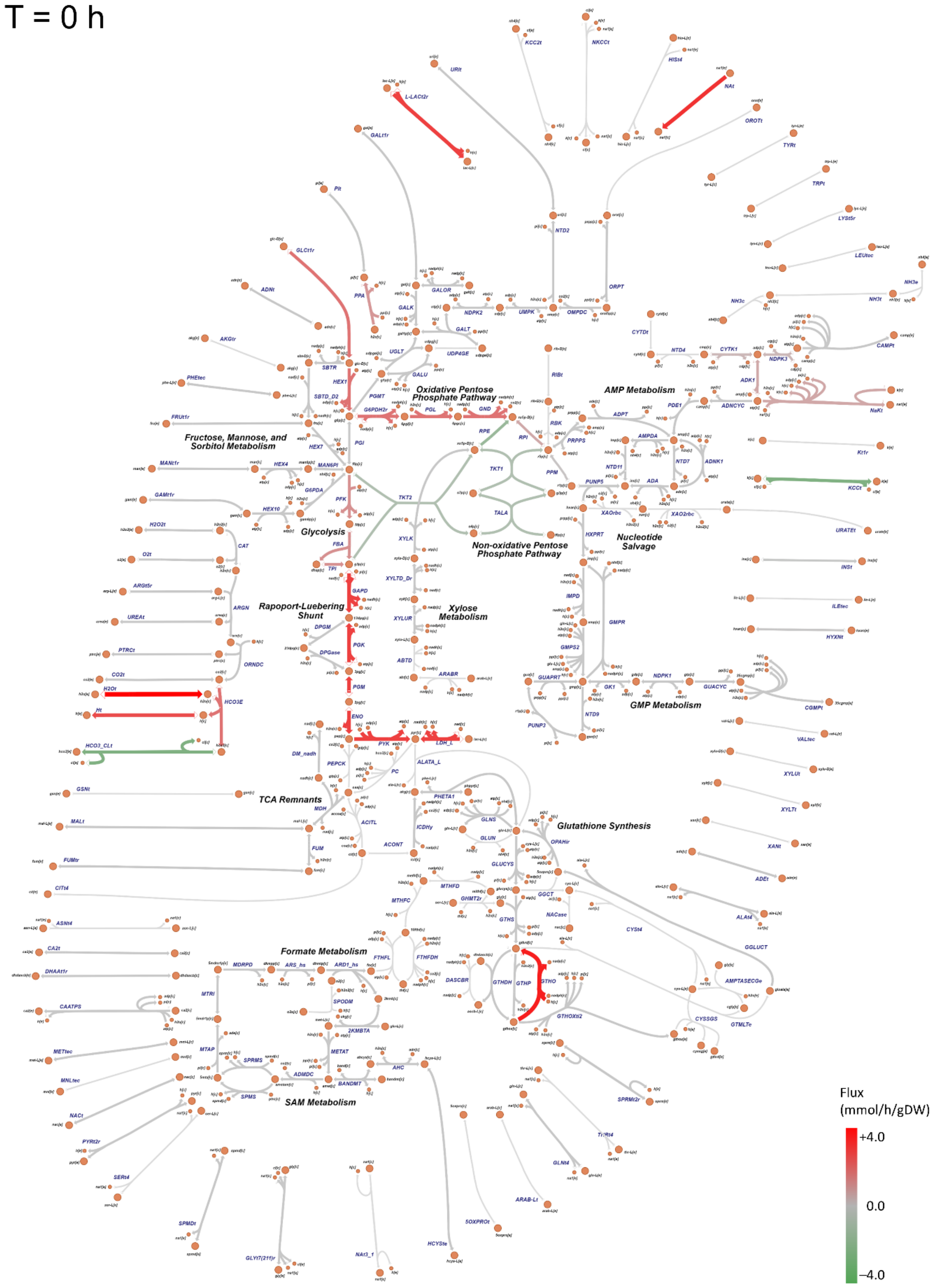

Figure S20: Overview of estimated metabolic fluxes at 8 h in red blood cells (RBC) maintained under ‘+Fos’ medium condition.

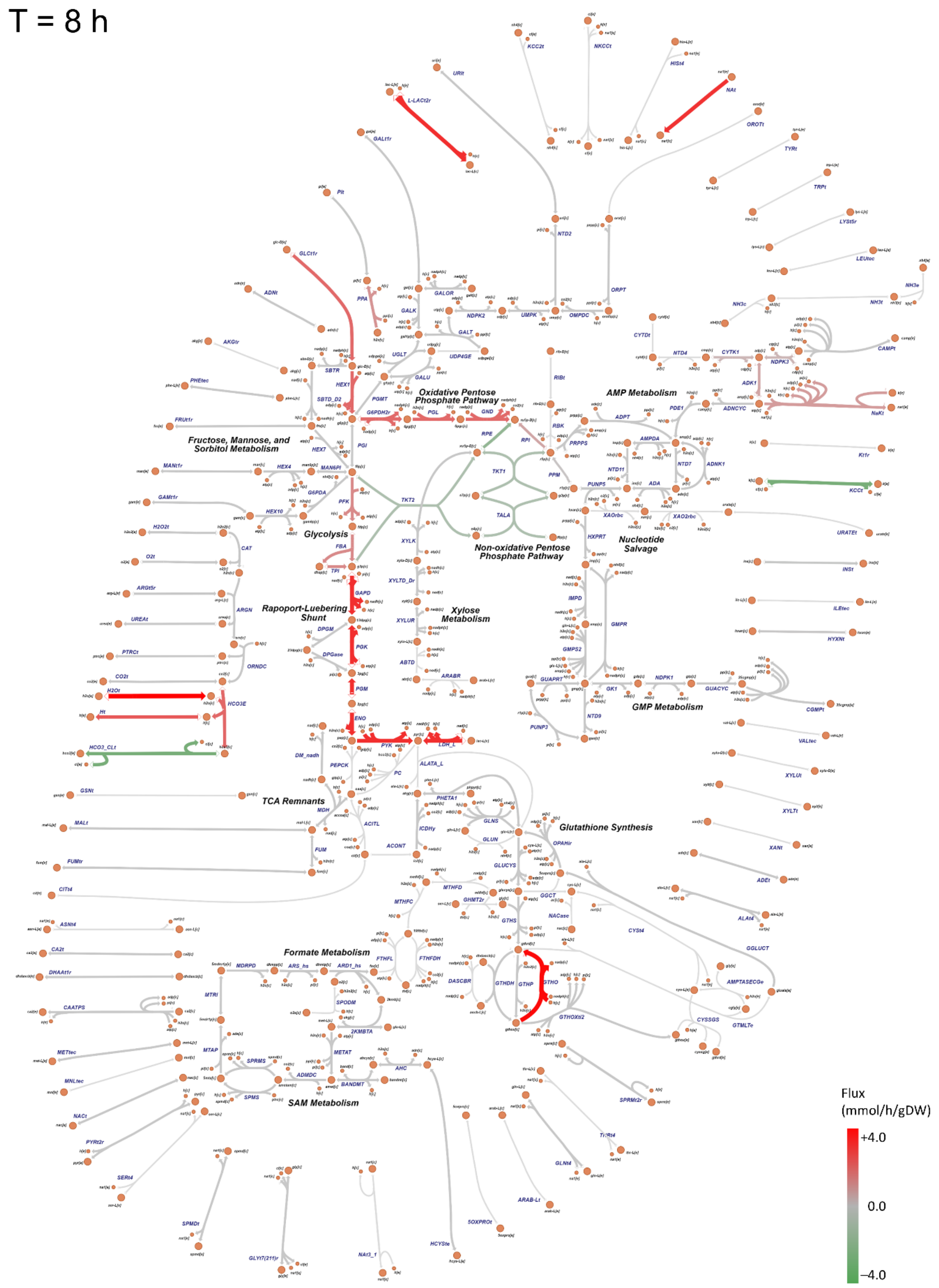

Figure S21: Overview of estimated metabolic fluxes at 16 h in red blood cells (RBC) maintained under ‘+Fos’ medium condition.

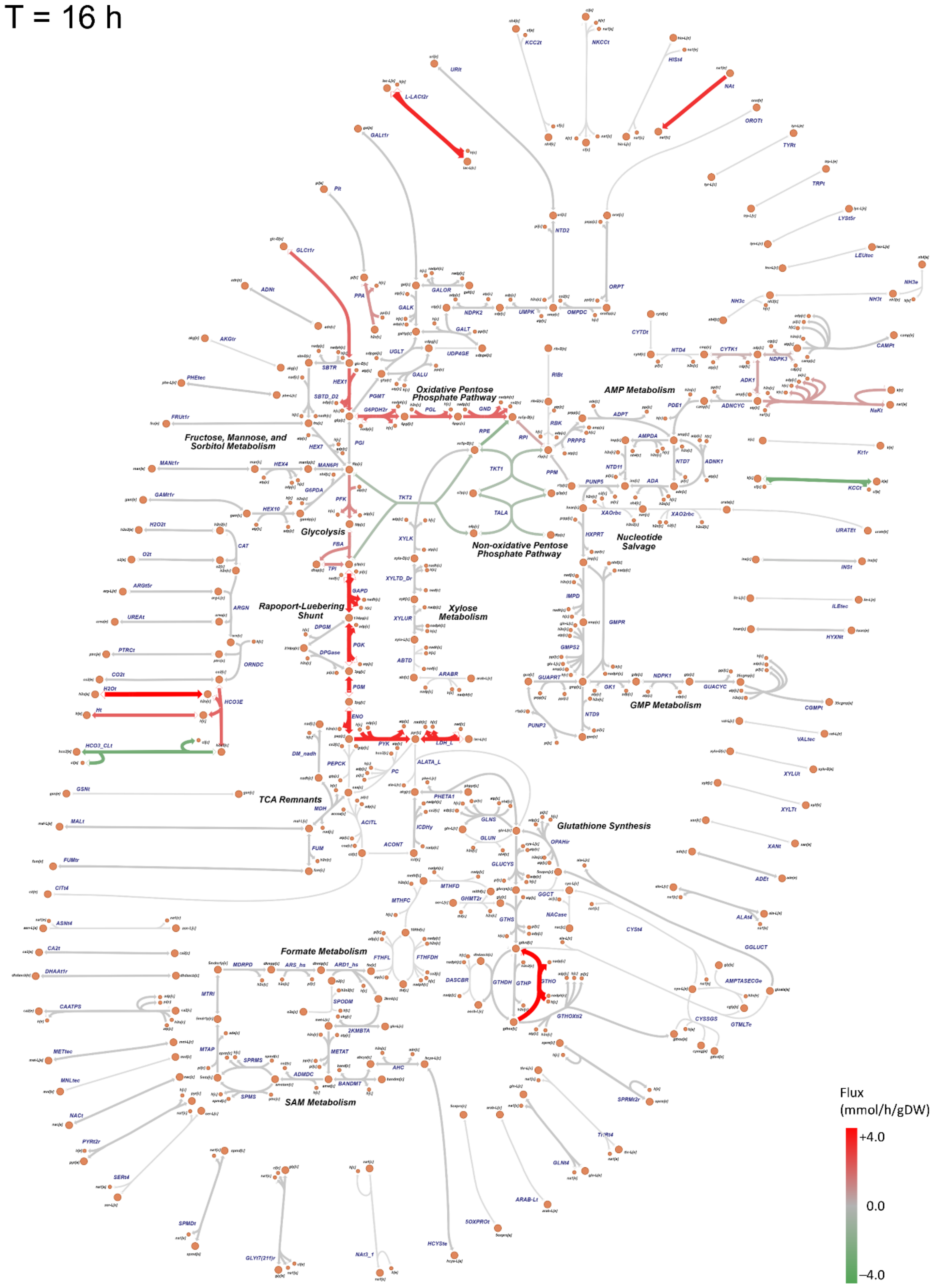

Figure S22: Overview of estimated metabolic fluxes at 24 h in red blood cells (RBC) maintained under '+Fos' medium condition.

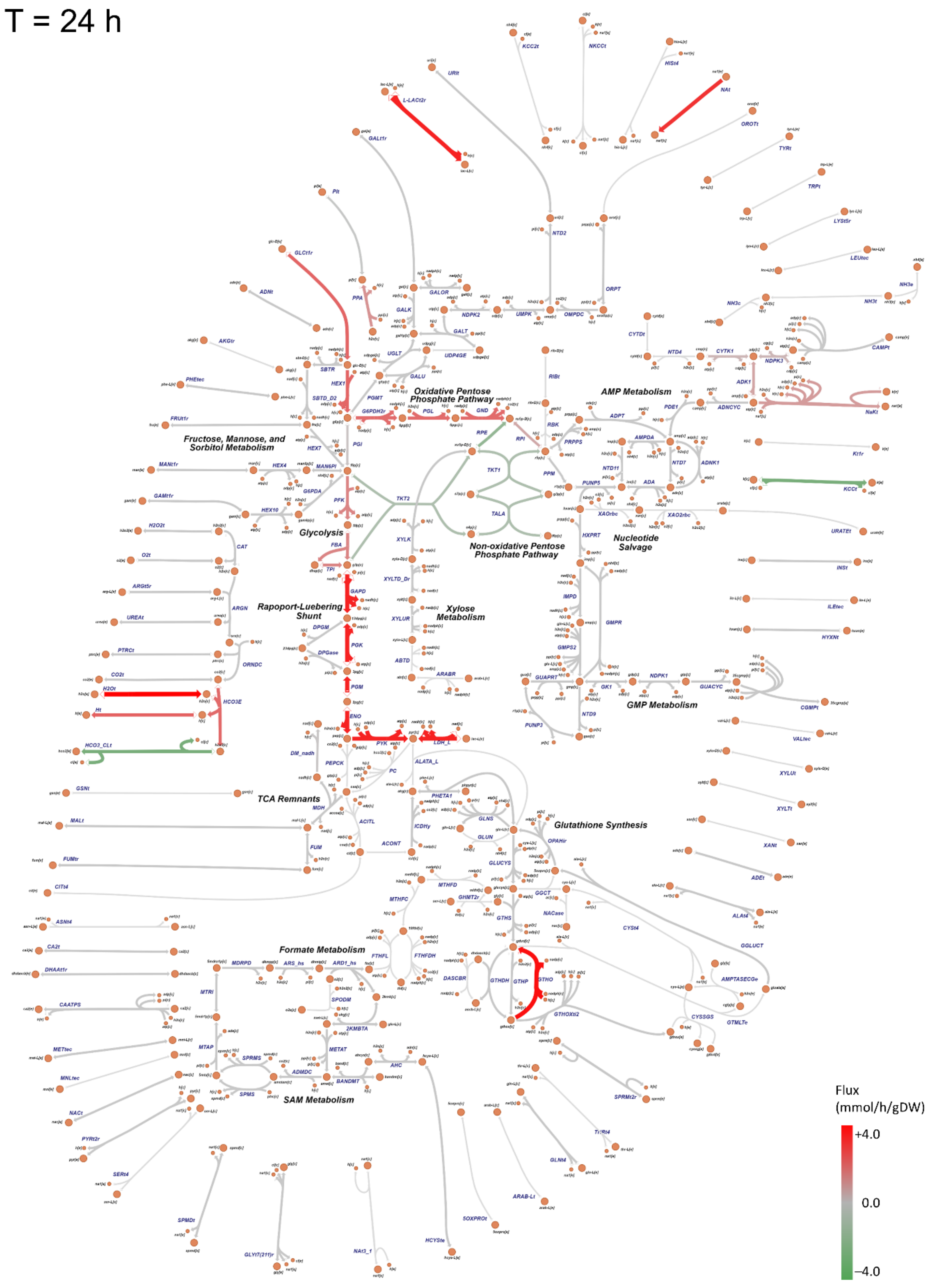

Figure S23: Overview of estimated metabolic fluxes at 32 h in red blood cells (RBC) maintained under '+Fos' medium condition.

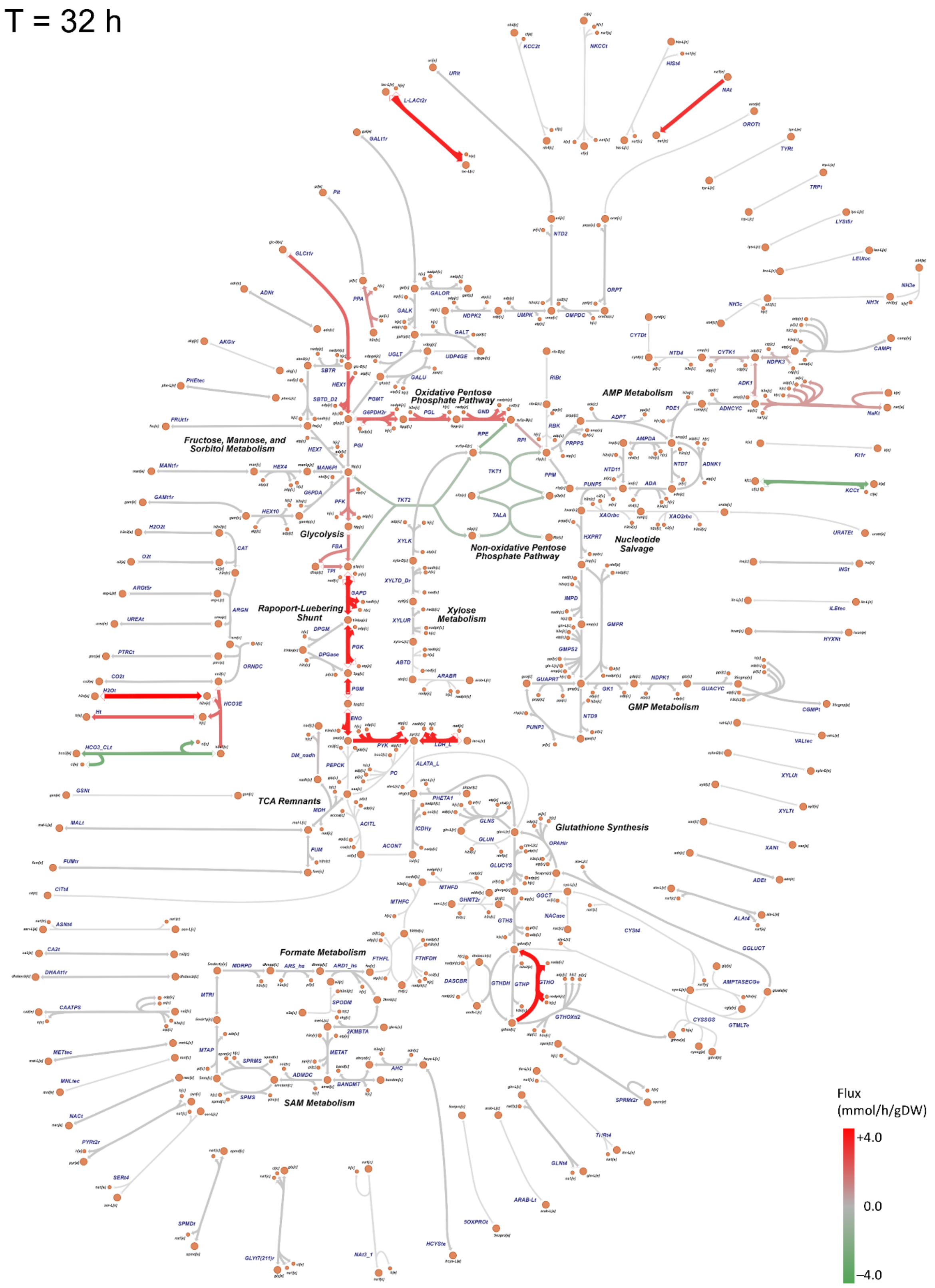

Figure S24: Overview of estimated metabolic fluxes at 40 h in red blood cells (RBC) maintained under '+Fos' medium condition.

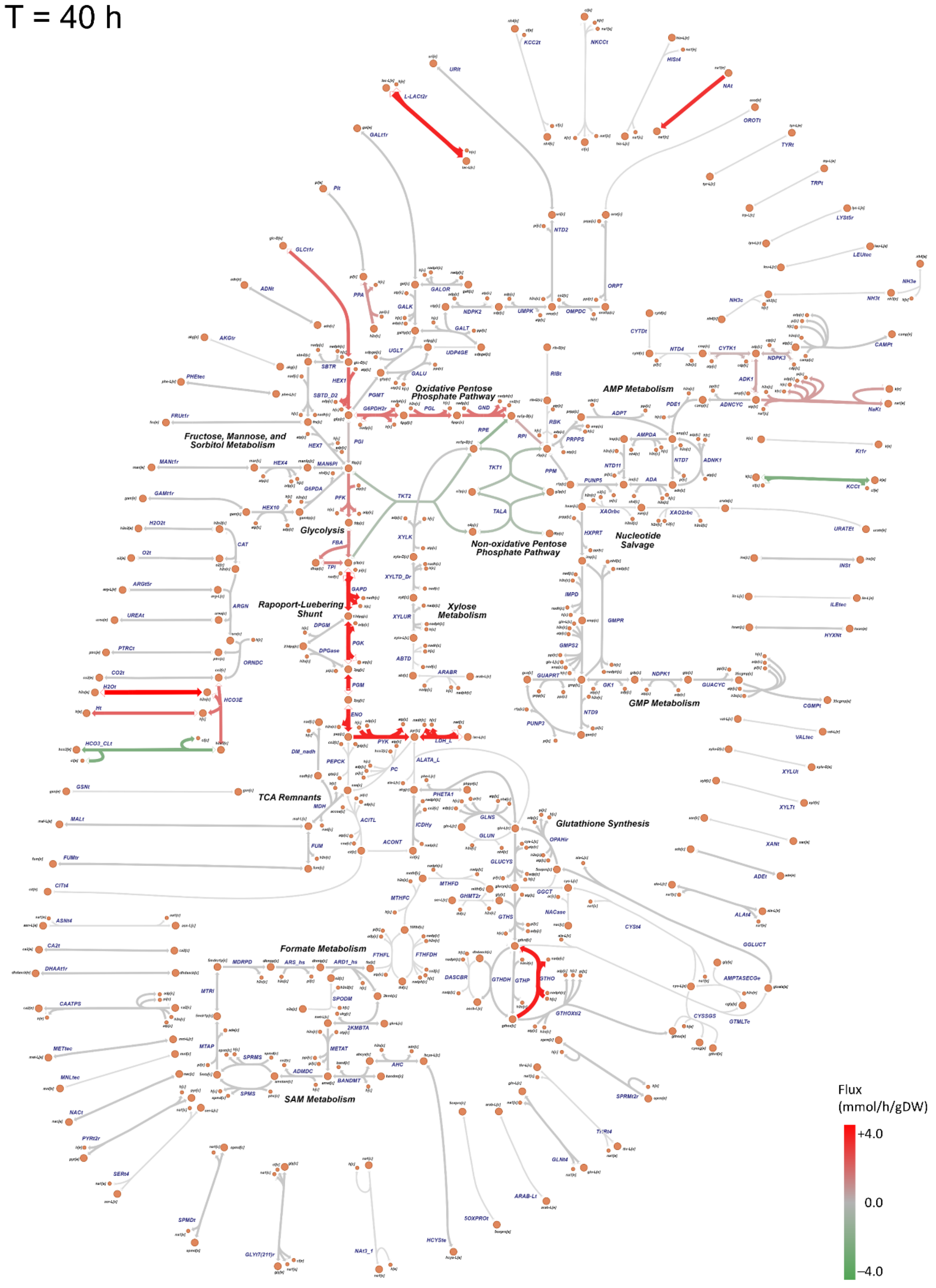

Figure S25: Overview of estimated metabolic fluxes at 0 h in red blood cells (RBC) maintained under ‘Pure 2’ medium condition.

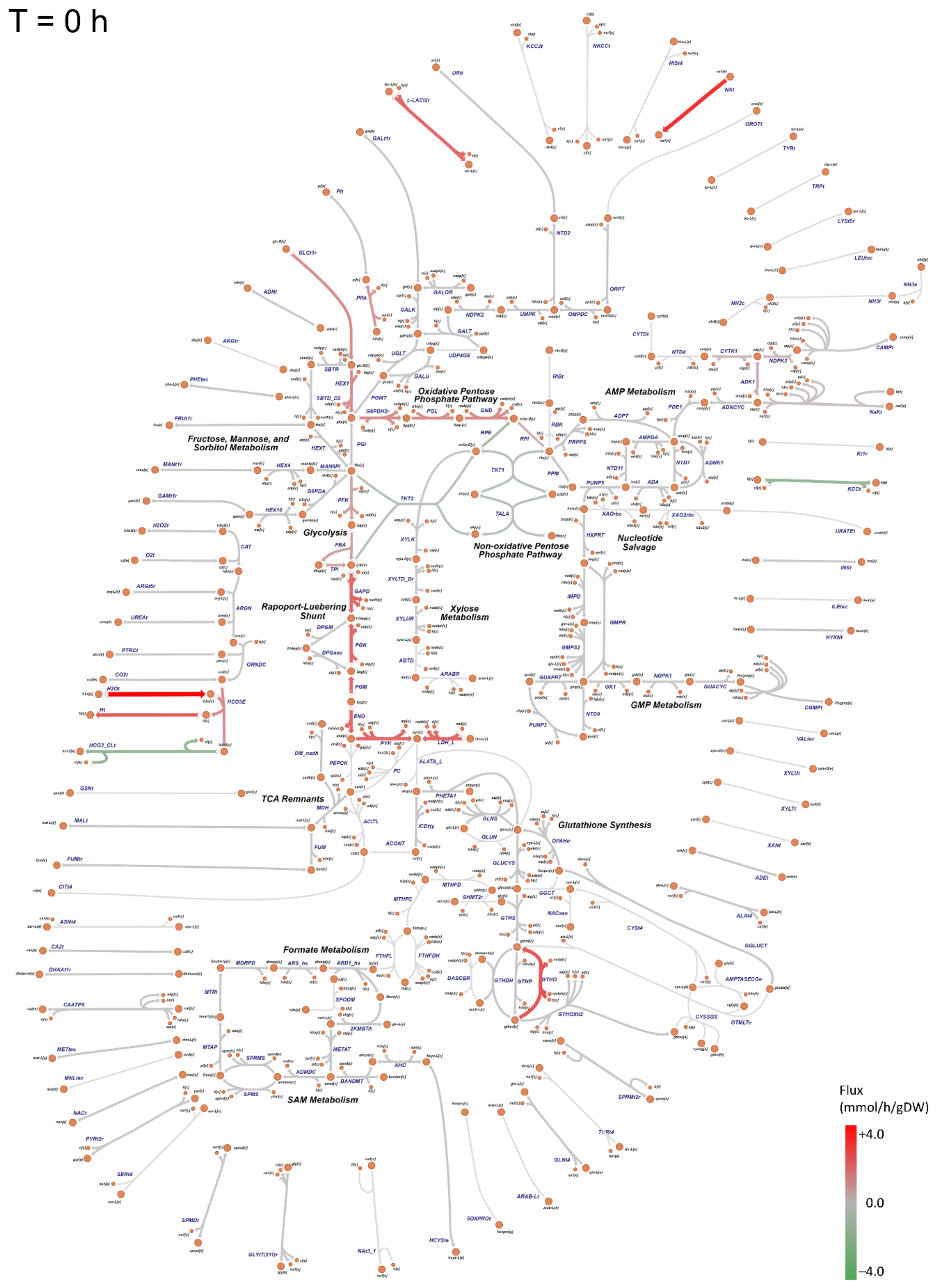

Figure S26: Overview of estimated metabolic fluxes at 8 h in red blood cells (RBC) maintained under ‘Pure 2’ medium condition.

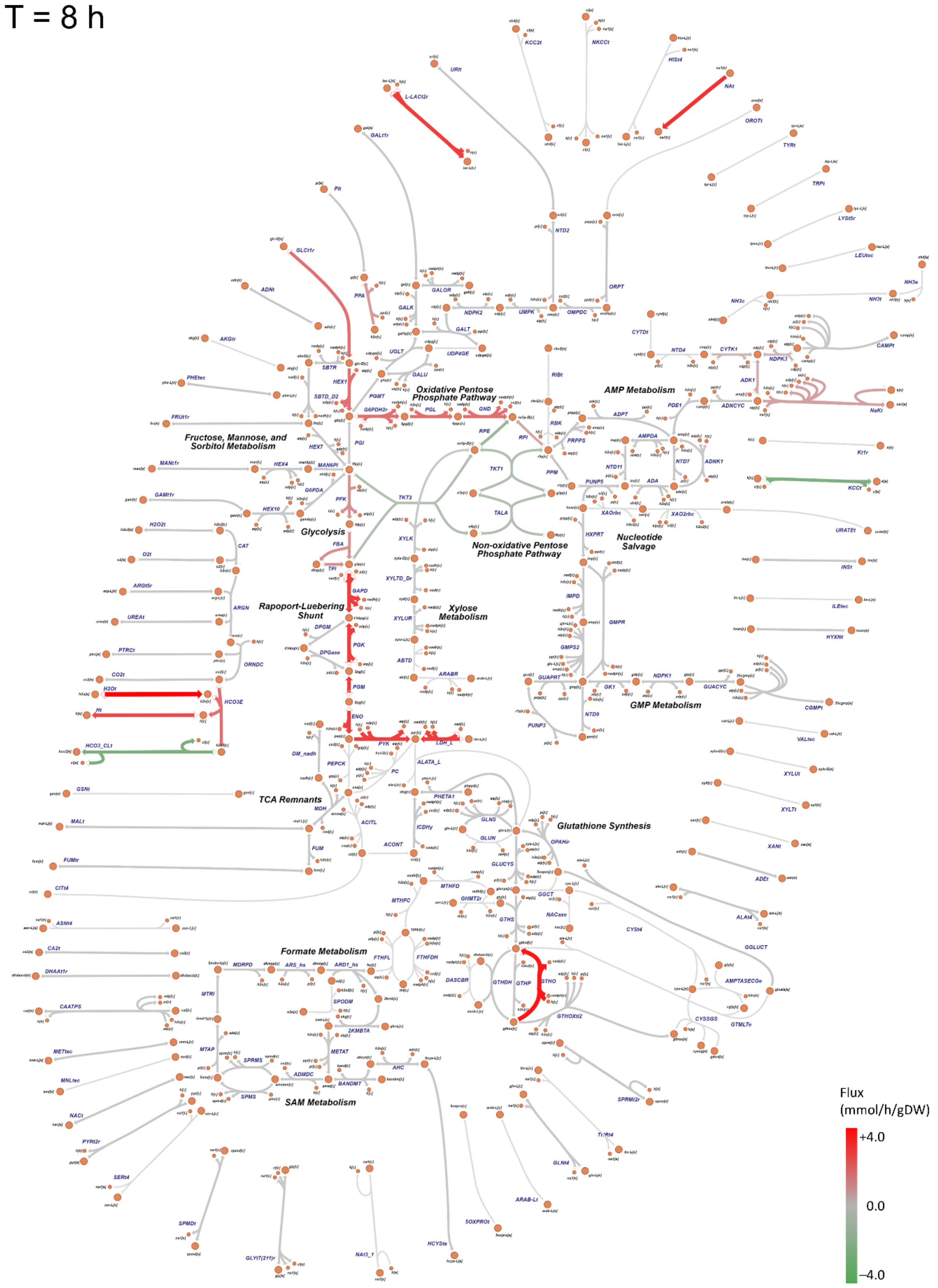

Figure S27: Overview of estimated metabolic fluxes at 16 h in red blood cells (RBC) maintained under ‘Pure 2’ medium condition.

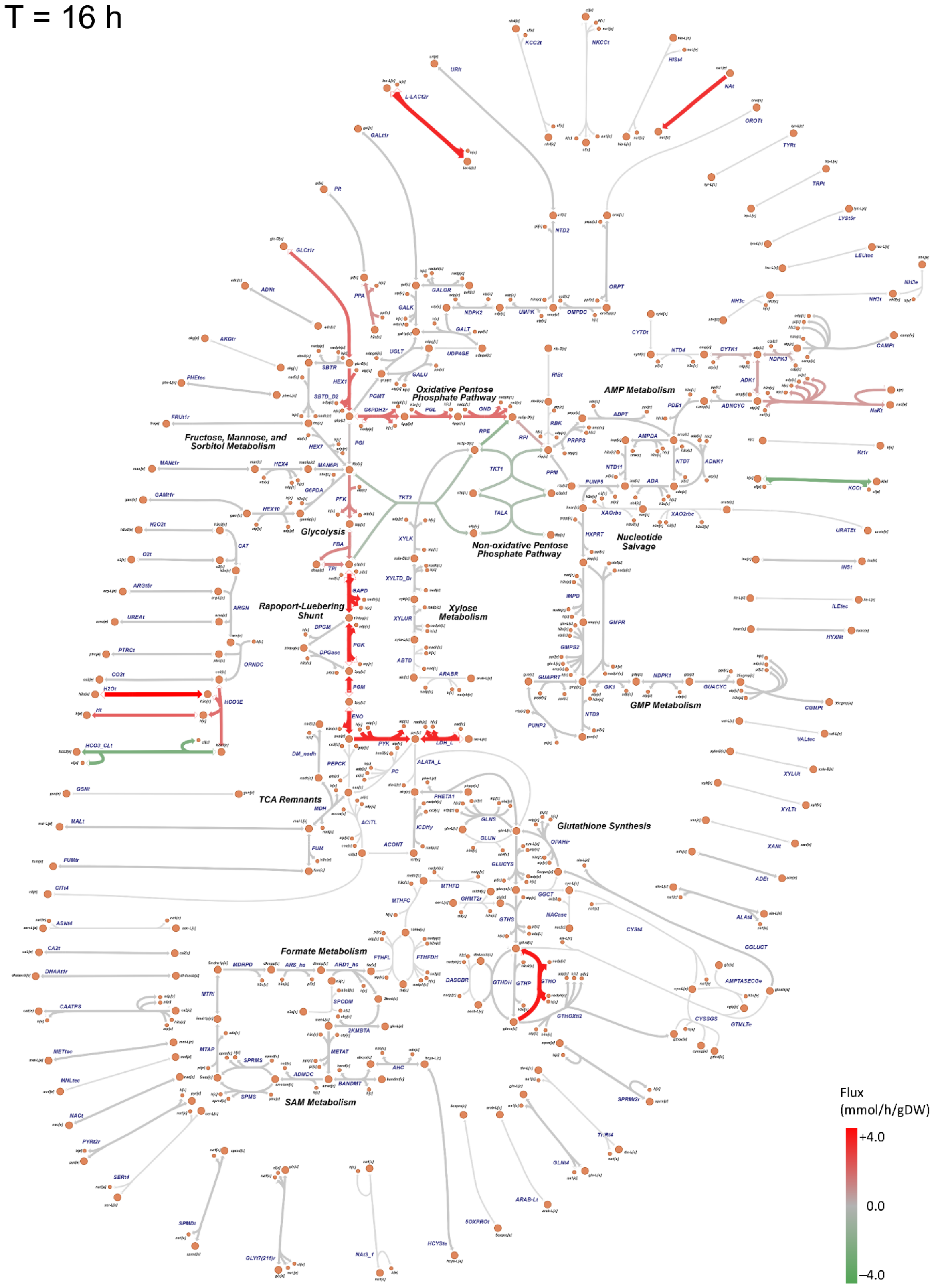

Figure S28: Overview of estimated metabolic fluxes at 24 h in red blood cells (RBC) maintained under ‘Pure 2’ medium condition.

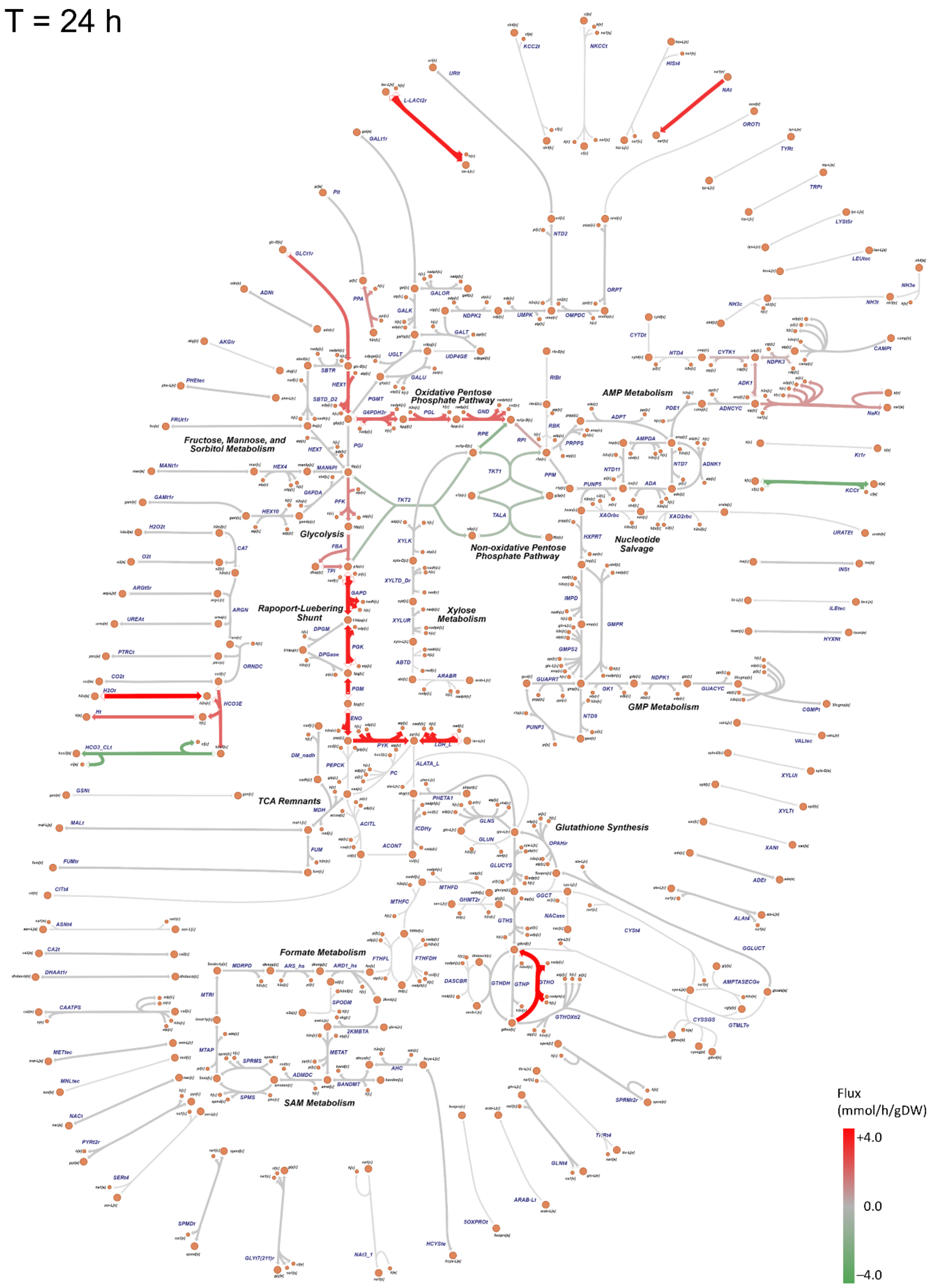

Figure S29: Overview of estimated metabolic fluxes at 32 h in red blood cells (RBC) maintained under ‘Pure 2’ medium condition.

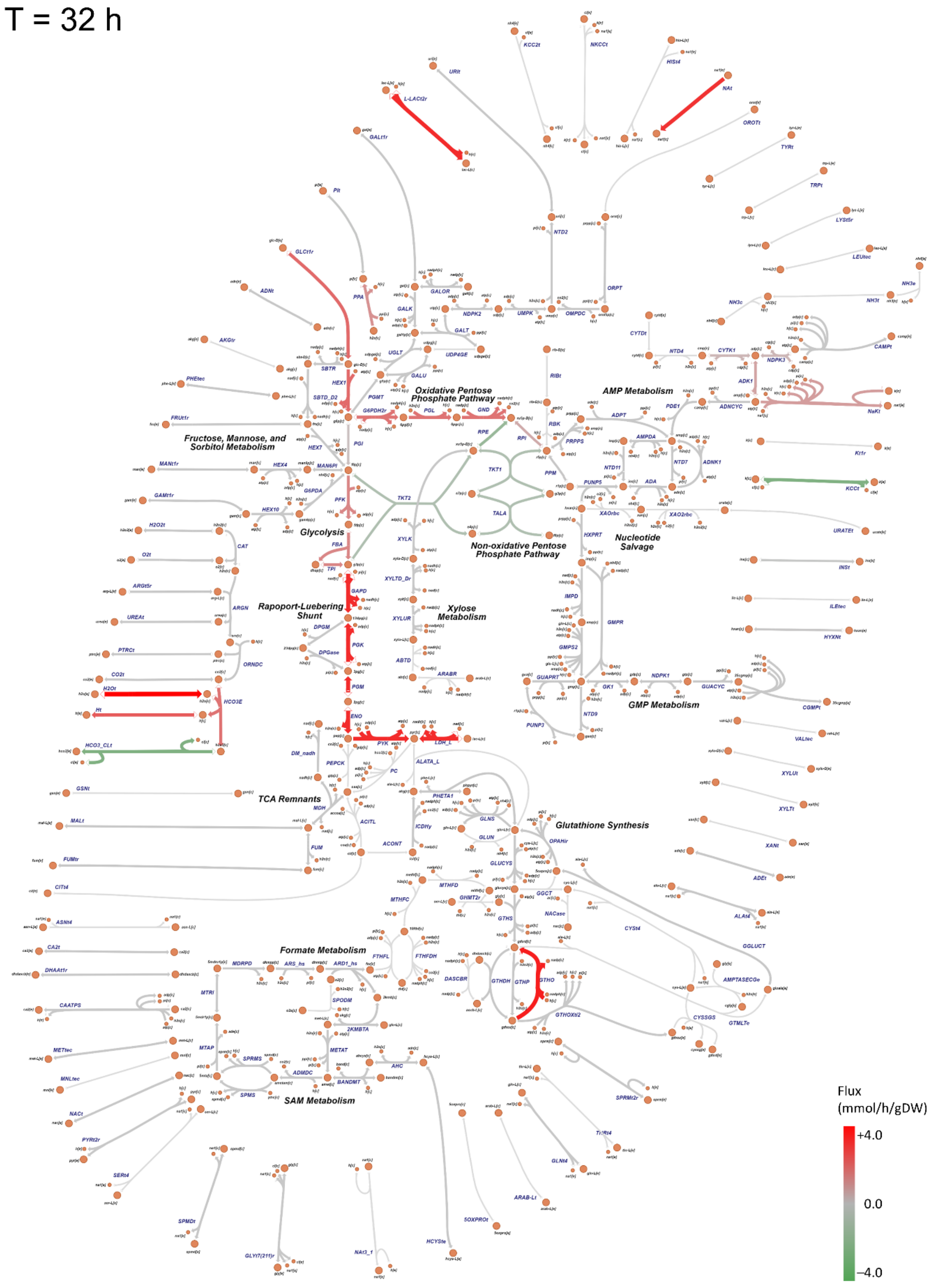

Figure S30: Overview of estimated metabolic fluxes at 40 h in red blood cells (RBC) maintained under 'Pure 2' medium condition.

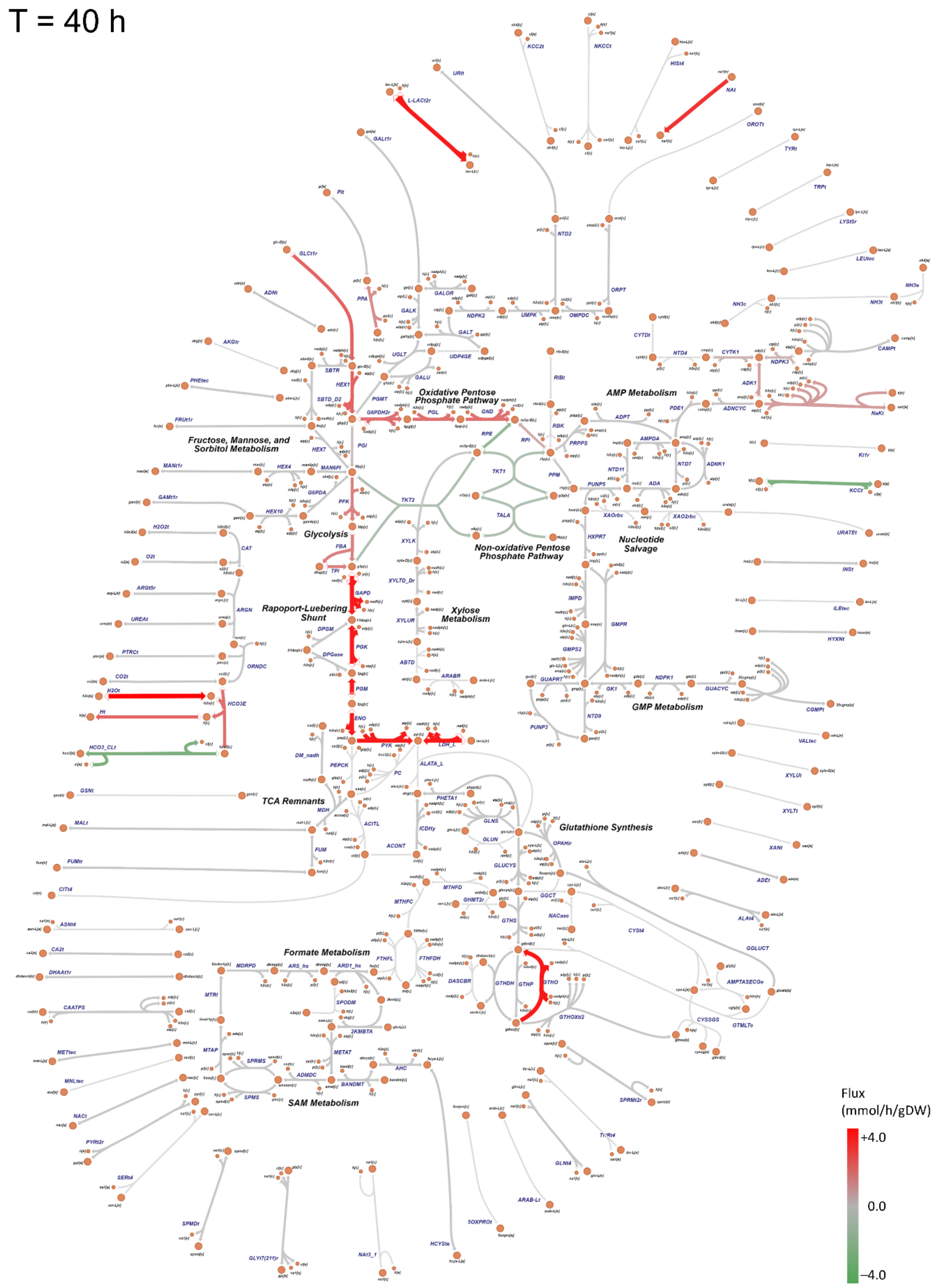

References

1. Olszewski KL, Morrisey JM, Wilinski D, Burns JM, Vaidya AB, Rabinowitz JD, Llinas M. 2009. Host-parasite interactions revealed by *Plasmodium falciparum* metabolomics. Cell Host Microbe 5:191-9.
2. Tewari SG, Rajaram K, Schyman P, Swift R, Reifman J, Prigge ST, Wallqvist A. 2019. Short-term metabolic adjustments in *Plasmodium falciparum* counter hypoxanthine deprivation at the expense of long-term viability. Malar J 18:86.
3. Heirendt L, Arreckx S, Pfau T, Mendoza SN, Richelle A, Heinken A, Haraldsdottir HS, Wachowiak J, Keating SM, Vlasov V, Magnusdottir S, Ng CY, Preciat G, Zagare A, Chan SHJ, Aurich MK, Clancy CM, Modamio J, Sauls JT, Noronha A, Bordbar A, Cousins B, El Assal DC, Valcarcel LV, Apaolaza I, Ghaderi S, Ahookhosh M, Ben Guebila M, Kostromins A, Sompairac N, Le HM, Ma D, Sun Y, Wang L, Yurkovich JT, Oliveira MAP, Vuong PT, El Assal LP, Kuperstein I, Zinovyev A, Hinton HS, Bryant WA, Aragon Artacho FJ, Planes FJ, Stalidzans E, Maass A, Vempala S, Hucka M, Saunders MA, Maranas CD, et al. 2019. Creation and analysis of biochemical constraint-based models using the COBRA Toolbox v.3.0. Nat Protoc 14:639-702.
4. Gatto C, Milanick M. 2009. Red blood cell Na pump: Insights from species differences. Blood Cells Mol Dis 42:192-200.
