# Supplementary material for: Inter-study and time-dependent variability of metabolite abundance in cultured red blood cells
Source: Malar J. 2021 Jul 2;20:299. doi: 10.1186/s12936-021-03780-5 (PMC8254254; doi:10.1186/s12936-021-03780-5)
Supplement: Supplementary file 5 — Additional file 5: Absolute differences of five high-magnitude fluxes within uRBCs of the five studies compared with their estimated fluxes in iRBCs. [file 12936_2021_3780_MOESM5_ESM.pdf]

Electronic Supplementary Material for the paper (Additional file 5):

# Inter-study and time-dependent variability of metabolite abundance in cultured red blood cells

By

Shivendra G. Tewari*, Krithika Rajaram, Russell P. Swift, Bobby Kwan, Jaques Reifman, Sean T. Prigge, and Anders Wallqvist*

***Corresponding authors. Emails: stewari@bhsai.org or sven.a.wallqvist.civ@mail.mil

This PDF file includes Tables S1–S5.

**Table S1**: Average (SD) of raw differences in glutathione oxidoreductase, GTHO, flux values between different conditions. We used published value of GTHO flux estimated in parasite-infected RBC (iRBC) [1] to compute the last column.

| | Pure 1 | -Hxn | +Mev | +Fos | Pure 2 | iRBC |
|---|---|---|---|---|---|---|
| Pure 1 | 0.00<br>(0.00) | 0.34<br>(0.24) | 0.46<br>(0.34) | 0.20<br>(0.21) | 0.43<br>(0.38) | 2.54<br>(0.31) |
| -Hxn | | 0.00<br>(0.00) | 0.20<br>(0.22) | 0.24<br>(0.19) | 0.54<br>(0.39) | 2.62<br>(0.37) |
| +Mev | | | 0.00<br>(0.00) | 0.26<br>(0.24) | 0.50<br>(0.38) | 2.66<br>(0.49) |
| +Fos | | | | 0.00<br>(0.00) | 0.34<br>(0.37) | 2.67<br>(0.21) |
| Pure 2 | | | | | 0.00<br>(0.00) | 2.45<br>(0.58) |
| iRBC | | | | | | 0.00<br>(0.00) |

**Table S2**: Average (SD) of raw differences in proton exchange, EX_h(e), flux values between different conditions. We used published value of EX_h(e) flux estimated in iRBC [1] to compute the last column.

| | Pure 1 | -Hxn | +Mev | +Fos | Pure 2 | iRBC |
|---|---|---|---|---|---|---|
| Pure 1 | 0.00 (0.00) | 0.14 (0.15) | 0.23 (0.19) | 0.11 (0.09) | 0.25 (0.38) | 57.8 (50.5) |
| -Hxn | | 0.00 (0.00) | 0.14 (0.17) | 0.08 (0.06) | 0.27 (0.49) | 57.7 (50.6) |
| +Mev | | | 0.00 (0.00) | 0.15 (0.16) | 0.34 (0.48) | 57.7 (50.6) |
| +Fos | | | | 0.00 (0.00) | 0.25 (0.45) | 57.7 (50.5) |
| Pure 2 | | | | | 0.00 (0.00) | 57.9 (50.3) |
| iRBC | | | | | | 0.00 (0.00) |

**Table S3**: Average (SD) of raw differences in h2o exchange, EX_h2o(e), flux values between different conditions. We used published value of EX_h2o(e) flux estimated in iRBC [1] to compute the last column.

| | Pure 1 | -Hxn | +Mev | +Fos | Pure 2 | iRBC |
|---|---|---|---|---|---|---|
| Pure 1 | 0.00 (0.00) | 0.01 (0.02) | 0.01 (0.03) | 0.00 (0.01) | 0.00 (0.01) | 13.7 (4.24) |
| -Hxn | | 0.00 (0.00) | 0.02 (0.03) | 0.01 (0.02) | 0.01 (0.02) | 13.7 (4.24) |
| +Mev | | | 0.00 (0.00) | 0.01 (0.03) | 0.01 (0.03) | 13.7 (4.25) |
| +Fos | | | | 0.00 (0.00) | 0.00 (0.00) | 13.6 (4.25) |
| Pure 2 | | | | | 0.00 (0.00) | 13.6 (4.25) |
| iRBC | | | | | | 0.00 (0.00) |

**Table S4**: Average (SD) of raw differences in L-lactate dehydrogenase, LDH_L, flux values between different conditions. We used published value of LDH_L flux estimated in iRBC [1] to compute the last column.

| | Pure 1 | -Hxn | +Mev | +Fos | Pure 2 | iRBC |
|---|---|---|---|---|---|---|
| Pure 1 | 0.00 (0.00) | 0.34 (0.38) | 0.44 (0.29) | 0.23 (0.24) | 0.18 (0.11) | 2.51 (0.52) |
| -Hxn | | 0.00 (0.00) | 0.23 (0.22) | 0.15 (0.12) | 0.35 (0.39) | 2.71 (0.18) |
| +Mev | | | 0.00 (0.00) | 0.23 (0.20) | 0.41 (0.35) | 2.62 (0.35) |
| +Fos | | | | 0.00 (0.00) | 0.25 (0.30) | 2.65 (0.23) |
| Pure 2 | | | | | 0.00 (0.00) | 2.48 (0.58) |
| iRBC | | | | | | 0.00 (0.00) |

**Table S5**: Average (SD) of raw differences in proton diffusion, Ht, flux values between different conditions. We used published value of Ht flux estimated in iRBC [1] to compute the last column.

| | Pure 1 | -Hxn | +Mev | +Fos | Pure 2 | iRBC |
|---|---|---|---|---|---|---|
| Pure 1 | 0.00 (0.00) | 0.15 (0.19) | 0.16 (0.19) | 0.10 (0.11) | 0.11 (0.15) | 9.16 (3.00) |
| -Hxn | | 0.00 (0.00) | 0.09 (0.06) | 0.08 (0.08) | 0.17 (0.20) | 9.09 (3.17) |
| +Mev | | | 0.00 (0.00) | 0.10 (0.07) | 0.20 (0.20) | 9.12 (3.17) |
| +Fos | | | | 0.00 (0.00) | 0.12 (0.15) | 9.14 (3.11) |
| Pure 2 | | | | | 0.00 (0.00) | 9.12 (3.12) |
| iRBC | | | | | | 0.00 (0.00) |

**Reference**


1. Wallqvist A, Fang X, Tewari SG, Ye P, Reifman J: **Metabolic host responses to malarial infection during the intraerythrocytic developmental cycle.** *BMC Syst Biol* 2016, **10:**58.
