# Supplementary material for: Inter-study and time-dependent variability of metabolite abundance in cultured red blood cells
Source: Malar J. 2021 Jul 2;20:299. doi: 10.1186/s12936-021-03780-5 (PMC8254254; doi:10.1186/s12936-021-03780-5)
Supplement: Supplementary file 6 — Additional file 6: Absolute differences of substantially varying fluxes, listed in Table 3, within uRBCs of the five studies compared with their estimated fluxes in iRBCs. [file 12936_2021_3780_MOESM6_ESM.pdf]

Electronic Supplementary Material for the paper (Additional file 6):

Inter-study and time-dependent variability of metabolite abundance in cultured red blood cells

By

Shivendra G. Tewari*, Krithika Rajaram, Russell P. Swift, Bobby Kwan, Jaques Reifman, Sean T. Prigge, and Anders Wallqvist*

*****Corresponding author. Email(s): stewari@bhsai.org or sven.a.wallqvist.civ@mail.mil

This PDF file includes Tables S1–S19.

Table S1: Average (SD) of raw differences in bicarbonate transport, HCO3_CLt, flux values between different conditions. We used published value of HCO3_CLt flux estimated in parasite-infected RBC (iRBC) [1] to compute the last column.

	**Pure 1**	**-Hxn**	**+Mev**	**+Fos**	**Pure 2**	**iRBC**
**Pure 1**	0.00 (0.00)	0.17 (0.12)	0.23 (0.17)	0.10 (0.10)	0.21 (0.20)	1.73 (0.15)
**-Hxn**		0.00 (0.00)	0.10 (0.11)	0.12 (0.10)	0.27 (0.20)	1.77 (0.18)
**+Mev**			0.00 (0.00)	0.13 (0.12)	0.25 (0.19)	1.79 (0.25)
**+Fos**				0.00 (0.00)	0.17 (0.19)	1.79 (0.11)
**Pure 2**					0.00 (0.00)	1.68 (0.29)
**iRBC**						0.00 (0.00)

Table S2: Average (SD) of raw differences in bicarbonate equilibration, HCO3E, flux values between different conditions. We used published value of HCO3E flux estimated in iRBC [1] to compute the last column.

	**Pure 1**	**-Hxn**	**+Mev**	**+Fos**	**Pure 2**	**iRBC**
**Pure 1**	0.00 (0.00)	0.17 (0.12)	0.23 (0.17)	0.10 (0.10)	0.21 (0.20)	1.73 (0.15)
**-Hxn**		0.00 (0.00)	0.10 (0.11)	0.12 (0.10)	0.27 (0.20)	1.77 (0.18)
**+Mev**			0.00 (0.00)	0.13 (0.12)	0.25 (0.19)	1.79 (0.25)
**+Fos**				0.00 (0.00)	0.17 (0.19)	1.79 (0.11)
**Pure 2**					0.00 (0.00)	1.68 (0.29)
**iRBC**						0.00 (0.00)

Table S3: Average (SD) of raw differences in glutathione oxidoreductase, GTHO, flux values between different conditions. We used published value of GTHO flux estimated in parasite-infected RBC (iRBC) [1] to compute the last column.

	**Pure 1**	**-Hxn**	**+Mev**	**+Fos**	**Pure 2**	**iRBC**
**Pure 1**	0.00 (0.00)	0.34 (0.24)	0.46 (0.34)	0.20 (0.21)	0.43 (0.38)	2.54 (0.31)
**-Hxn**		0.00 (0.00)	0.20 (0.22)	0.24 (0.19)	0.54 (0.39)	2.62 (0.37)
**+Mev**			0.00 (0.00)	0.26 (0.24)	0.50 (0.38)	2.66 (0.49)
**+Fos**				0.00 (0.00)	0.34 (0.37)	2.67 (0.21)
**Pure 2**					0.00 (0.00)	2.45 (0.58)
**iRBC**						0.00 (0.00)

Table S4: Average (SD) of raw differences in enolase, ENO, flux values between different conditions. We used published value of ENO flux estimated in iRBC [1] to compute the last column.

	Pure 1	-Hxn	+Mev	+Fos	Pure 2	iRBC
**Pure 1**	0.00 (0.00)	0.27 (0.30)	0.42 (0.22)	0.17 (0.14)	0.21 (0.14)	2.49 (0.47)
**-Hxn**		0.00 (0.00)	0.19 (0.20)	0.15 (0.17)	0.35 (0.33)	2.63 (0.26)
**+Mev**			0.00 (0.00)	0.26 (0.13)	0.42 (0.37)	2.59 (0.35)
**+Fos**				0.00 (0.00)	0.27 (0.27)	2.55 (0.27)
**Pure 2**					0.00 (0.00)	2.41 (0.61)
**iRBC**						0.00 (0.00)

Table S5: Average (SD) of raw differences in glyceraldehyde-3-phosphate dehydrogenase, GAPD, flux values between different conditions. We used published value of GAPD flux estimated in iRBC [1] to compute the last column.

	**Pure 1**	**-Hxn**	**+Mev**	**+Fos**	**Pure 2**	**iRBC**
**Pure 1**	0.00 (0.00)	0.27 (0.30)	0.42 (0.22)	0.17 (0.14)	0.21 (0.14)	2.45 (0.47)
**-Hxn**		0.00 (0.00)	0.19 (0.20)	0.15 (0.17)	0.35 (0.33)	2.60 (0.26)
**+Mev**			0.00 (0.00)	0.26 (0.13)	0.42 (0.37)	2.55 (0.35)
**+Fos**				0.00 (0.00)	0.27 (0.27)	2.52 (0.27)
**Pure 2**					0.00 (0.00)	2.38 (0.61)
**iRBC**						0.00 (0.00)

Table S6: Average (SD) of raw differences in glucose transport, GLCt1r, flux values between different conditions. We used published value of GLCt1r flux estimated in iRBC [1] to compute the last column.

	**Pure 1**	**-Hxn**	**+Mev**	**+Fos**	**Pure 2**	**iRBC**
**Pure 1**	0.00 (0.00)	0.14 (0.14)	0.20 (0.15)	0.08 (0.04)	0.17 (0.16)	34.36 (27.09)
**-Hxn**		0.00 (0.00)	0.12 (0.10)	0.08 (0.09)	0.24 (0.23)	34.30 (27.24)
**+Mev**			0.00 (0.00)	0.13 (0.11)	0.25 (0.21)	34.34 (27.22)
**+Fos**				0.00 (0.00)	0.18 (0.18)	34.34 (27.14)
**Pure 2**					0.00 (0.00)	34.44 (27.00)
**iRBC**						0.00 (0.00)

Table S7: Average (SD) of raw differences in L-lactate reversible transport, L-LACt2r, flux values between different conditions. We used published value of L-LACt2r flux estimated in iRBC [1] to compute the last column.

	**Pure 1**	**-Hxn**	**+Mev**	**+Fos**	**Pure 2**	**iRBC**
**Pure 1**	0.00 (0.00)	0.27 (0.30)	0.42 (0.22)	0.17 (0.14)	0.21 (0.14)	66.58 (53.31)
**-Hxn**		0.00 (0.00)	0.19 (0.20)	0.15 (0.17)	0.35 (0.33)	66.44 (53.64)
**+Mev**			0.00 (0.00)	0.26 (0.13)	0.42 (0.37)	66.48 (53.65)
**+Fos**				0.00 (0.00)	0.27 (0.27)	66.51 (53.47)
**Pure 2**					0.00 (0.00)	66.65 (53.29)
**iRBC**						0.00 (0.00)

Table S8: Average (SD) of raw differences in L-lactate dehydrogenase, LDH_L, flux values between different conditions. We used published value of LDH_L flux estimated in iRBC [1] to compute the last column.

	**Pure 1**	**-Hxn**	**+Mev**	**+Fos**	**Pure 2**	**iRBC**
**Pure 1**	0.00 (0.00)	0.34 (0.38)	0.44 (0.29)	0.23 (0.24)	0.18 (0.11)	2.51 (0.52)
**-Hxn**		0.00 (0.00)	0.23 (0.22)	0.15 (0.12)	0.35 (0.39)	2.71 (0.18)
**+Mev**			0.00 (0.00)	0.23 (0.20)	0.41 (0.35)	2.62 (0.35)
**+Fos**				0.00 (0.00)	0.25 (0.30)	2.65 (0.23)
**Pure 2**					0.00 (0.00)	2.48 (0.58)
**iRBC**						0.00 (0.00)

Table S9: Average (SD) of raw differences in phosphoglycerate kinase, PGK, flux values between different conditions. We used published value of PGK flux estimated in iRBC [1] to compute the last column.

	**Pure 1**	**-Hxn**	**+Mev**	**+Fos**	**Pure 2**	**iRBC**
**Pure 1**	0.00 (0.00)	0.18 (0.19)	0.17 (0.12)	0.17 (0.18)	0.06 (0.03)	0.81 (0.46)
**-Hxn**		0.00 (0.00)	0.10 (0.10)	0.03 (0.03)	0.17 (0.15)	0.91 (0.22)
**+Mev**			0.00 (0.00)	0.11 (0.09)	0.18 (0.07)	0.89 (0.30)
**+Fos**				0.00 (0.00)	0.15 (0.14)	0.91 (0.23)
**Pure 2**					0.00 (0.00)	0.82 (0.41)
**iRBC**						0.00 (0.00)

Table S10: Average (SD) of raw differences in phosphoglycerate mutase, PGM, flux values between different conditions. We used published value of PGM flux estimated in iRBC [1] to compute the last column.

	**Pure 1**	**-Hxn**	**+Mev**	**+Fos**	**Pure 2**	**iRBC**
**Pure 1**	0.00 (0.00)	0.27 (0.30)	0.42 (0.22)	0.17 (0.14)	0.21 (0.14)	2.49 (0.47)
**-Hxn**		0.00 (0.00)	0.19 (0.20)	0.15 (0.17)	0.35 (0.33)	2.63 (0.26)
**+Mev**			0.00 (0.00)	0.26 (0.13)	0.42 (0.37)	2.59 (0.35)
**+Fos**				0.00 (0.00)	0.27 (0.27)	2.55 (0.27)
**Pure 2**					0.00 (0.00)	2.41 (0.61)
**iRBC**						0.00 (0.00)

Table S11: Average (SD) of raw differences in pyruvate kinase, PYK, flux values between different conditions. We used published value of PYK flux estimated in iRBC [1] to compute the last column.

	**Pure 1**	**-Hxn**	**+Mev**	**+Fos**	**Pure 2**	**iRBC**
**Pure 1**	0.00 (0.00)	0.27 (0.30)	0.42 (0.22)	0.17 (0.14)	0.21 (0.14)	2.49 (0.47)
**-Hxn**		0.00 (0.00)	0.19 (0.20)	0.15 (0.17)	0.35 (0.33)	2.63 (0.26)
**+Mev**			0.00 (0.00)	0.26 (0.13)	0.42 (0.37)	2.59 (0.35)
**+Fos**				0.00 (0.00)	0.27 (0.27)	2.55 (0.27)
**Pure 2**					0.00 (0.00)	2.41 (0.61)
**iRBC**						0.00 (0.00)

Table S12: Average (SD) of raw differences in hexokinase, HEX1, flux values between different conditions. We used published value of HEX1 flux estimated in iRBC [1] to compute the last column.

	**Pure 1**	**-Hxn**	**+Mev**	**+Fos**	**Pure 2**	**iRBC**
**Pure 1**	0.00 (0.00)	0.14 (0.14)	0.20 (0.15)	0.08 (0.04)	0.17 (0.16)	1.22 (0.17)
**-Hxn**		0.00 (0.00)	0.12 (0.10)	0.08 (0.09)	0.24 (0.23)	1.27 (0.10)
**+Mev**			0.00 (0.00)	0.13 (0.11)	0.25 (0.21)	1.23 (0.21)
**+Fos**				0.00 (0.00)	0.18 (0.18)	1.24 (0.11)
**Pure 2**					0.00 (0.00)	1.13 (0.34)
**iRBC**						0.00 (0.00)

Table S13: Average (SD) of raw differences in K^+–Cl^- symporter, KCCt, flux values between different conditions. We used published value of KCCt flux estimated in iRBC [1] to compute the last column.

	**Pure 1**	**-Hxn**	**+Mev**	**+Fos**	**Pure 2**	**iRBC**
**Pure 1**	0.00 (0.00)	0.17 (0.12)	0.23 (0.17)	0.10 (0.10)	0.21 (0.20)	1.73 (0.15)
**-Hxn**		0.00 (0.00)	0.10 (0.11)	0.12 (0.10)	0.27 (0.20)	1.77 (0.18)
**+Mev**			0.00 (0.00)	0.13 (0.12)	0.25 (0.19)	1.79 (0.25)
**+Fos**				0.00 (0.00)	0.17 (0.19)	1.79 (0.11)
**Pure 2**					0.00 (0.00)	1.68 (0.29)
**iRBC**						0.00 (0.00)

Table S14: Average (SD) of raw differences in Na^+/K^+ ATPase, NaKt, flux values between different conditions. We used published value of NaKt flux estimated in iRBC [1] to compute the last column.

	**Pure 1**	**-Hxn**	**+Mev**	**+Fos**	**Pure 2**	**iRBC**
**Pure 1**	0.00 (0.00)	0.22 (0.26)	0.21 (0.26)	0.18 (0.23)	0.14 (0.15)	0.71 (0.31)
**-Hxn**		0.00 (0.00)	0.09 (0.11)	0.08 (0.05)	0.13 (0.10)	0.87 (0.09)
**+Mev**			0.00 (0.00)	0.11 (0.05)	0.12 (0.10)	0.84 (0.13)
**+Fos**				0.00 (0.00)	0.06 (0.07)	0.84 (0.05)
**Pure 2**					0.00 (0.00)	0.82 (0.14)
**iRBC**						0.00 (0.00)

Table S15: Average (SD) of raw differences in inorganic diphosphatase, PPA, flux values between different conditions. We used published value of PPA flux estimated in iRBC [1] to compute the last column. Note that the differences between PPA fluxes occur at third and fourth decimal places, which are not visible due to the rounding off at the second decimal place.

	**Pure 1**	**-Hxn**	**+Mev**	**+Fos**	**Pure 2**	**iRBC**
**Pure 1**	0.00 (0.00)	0.01 (0.01)	0.01 (0.01)	0.01 (0.02)	0.01 (0.01)	0.02 (0.01)
**-Hxn**		0.00 (0.00)	0.01 (0.02)	0.01 (0.01)	0.00 (0.01)	0.02 (0.01)
**+Mev**			0.00 (0.00)	0.01 (0.03)	0.01 (0.01)	0.02 (0.01)
**+Fos**				0.00 (0.00)	0.01 (0.02)	0.03 (0.01)
**Pure 2**					0.00 (0.00)	0.02 (0.01)
**iRBC**						0.00 (0.00)

Table S16: Average (SD) of raw differences in glucose 6-phosphate dehydrogenase, G6PDH2r, flux values between different conditions. We used published value of G6PDH2r flux estimated in iRBC [1] to compute the last column.

	**Pure 1**	**-Hxn**	**+Mev**	**+Fos**	**Pure 2**	**iRBC**
**Pure 1**	0.00 (0.00)	0.17 (0.12)	0.23 (0.17)	0.10 (0.10)	0.21 (0.19)	1.27 (0.16)
**-Hxn**		0.00 (0.00)	0.10 (0.11)	0.12 (0.10)	0.27 (0.20)	1.31 (0.18)
**+Mev**			0.00 (0.00)	0.13 (0.12)	0.25 (0.19)	1.33 (0.25)
**+Fos**				0.00 (0.00)	0.17 (0.19)	1.34 (0.11)
**Pure 2**					0.00 (0.00)	1.22 (0.29)
**iRBC**						0.00 (0.00)

Table S17: Average (SD) of raw differences in phosphogluconate dehydrogenase, GND, flux values between different conditions. We used published value of GND flux estimated in iRBC [1] to compute the last column.

	**Pure 1**	**-Hxn**	**+Mev**	**+Fos**	**Pure 2**	**iRBC**
**Pure 1**	0.00 (0.00)	0.17 (0.12)	0.23 (0.17)	0.10 (0.10)	0.21 (0.19)	1.27 (0.16)
**-Hxn**		0.00 (0.00)	0.10 (0.11)	0.12 (0.10)	0.27 (0.20)	1.31 (0.18)
**+Mev**			0.00 (0.00)	0.13 (0.12)	0.25 (0.19)	1.33 (0.25)
**+Fos**				0.00 (0.00)	0.17 (0.19)	1.34 (0.11)
**Pure 2**					0.00 (0.00)	1.22 (0.29)
**iRBC**						0.00 (0.00)

Table S18: Average (SD) of raw differences in 6-phosphogluconolactonase, PGL, flux values between different conditions. We used published value of PGL flux estimated in iRBC [1] to compute the last column.

	**Pure 1**	**-Hxn**	**+Mev**	**+Fos**	**Pure 2**	**iRBC**
**Pure 1**	0.00 (0.00)	0.17 (0.12)	0.23 (0.17)	0.10 (0.10)	0.21 (0.19)	1.27 (0.16)
**-Hxn**		0.00 (0.00)	0.10 (0.11)	0.12 (0.10)	0.27 (0.20)	1.31 (0.18)
**+Mev**			0.00 (0.00)	0.13 (0.12)	0.25 (0.19)	1.33 (0.25)
**+Fos**				0.00 (0.00)	0.17 (0.19)	1.34 (0.11)
**Pure 2**					0.00 (0.00)	1.22 (0.29)
**iRBC**						0.00 (0.00)

Table S19: Average (SD) of raw differences in ribulose 5-phosphate 3-epimerase, RPE, flux values between different conditions. We used published value of RPE flux estimated in iRBC [1] to compute the last column.

	**Pure 1**	**-Hxn**	**+Mev**	**+Fos**	**Pure 2**	**iRBC**
**Pure 1**	0.00 (0.00)	0.11 (0.09)	0.16 (0.12)	0.06 (0.07)	0.14 (0.12)	1.05 (0.11)
**-Hxn**		0.00 (0.00)	0.07 (0.08)	0.08 (0.07)	0.18 (0.13)	1.08 (0.12)
**+Mev**			0.00 (0.00)	0.10 (0.07)	0.17 (0.13)	1.10 (0.16)
**+Fos**				0.00 (0.00)	0.10 (0.11)	1.09 (0.09)
**Pure 2**					0.00 (0.00)	1.03 (0.21)
**iRBC**						0.00 (0.00)

Reference

1. Wallqvist A, Fang X, Tewari SG, Ye P, Reifman J: **Metabolic host responses to malarial infection during the intraerythrocytic developmental cycle.** *BMC Syst Biol* 2016, **10:**58.
